# *miR-154* Influences HNSCC Development and Progression through Regulation of the Epithelial-to-Mesenchymal Transition Process and Could Be Used as a Potential Biomarker

**DOI:** 10.3390/biomedicines9121894

**Published:** 2021-12-13

**Authors:** Weronika Tomaszewska, Joanna Kozłowska-Masłoń, Dawid Baranowski, Anna Perkowska, Sandra Szałkowska, Urszula Kazimierczak, Patricia Severino, Katarzyna Lamperska, Tomasz Kolenda

**Affiliations:** 1 Department of Cancer Immunology, Chair of Medical Biotechnology, Poznan University of Medical Sciences, 8 Rokietnicka Street, 60-806 Poznan, Poland; airiarn@gmail.com (D.B.); anqq95@gmail.com (A.P.); sandra.j.szalkowska@gmail.com (S.S.); ukazimierczak@ump.edu.pl (U.K.); 2 Laboratory of Cancer Genetics, Greater Poland Cancer Centre, Garbary 15, 61-866 Poznan, Poland; joanna.kozlowska@wco.pl (J.K.-M.); katarzyna.lamperska@wco.pl (K.L.); 3 Research and Implementation Unit, Greater Poland Cancer Centre, Garbary 15, 61-866 Poznan, Poland; 4 Faculty of Biology, Institute of Human Biology and Evolution, Adam Mickiewicz University, Uniwersytetu Poznańskiego 6, 61-614 Poznań, Poland; 5 Centro de Pesquisa Experimental, Albert Einstein Research and Education Institute, Hospital Israelita Albert Einstein, Av. Albert Einstein, 627-Jardim Leonor, São Paulo 05652-900, SP, Brazil; patricia.severino@einstein.br

**Keywords:** *miR-154*, microRNA, ncRNA, HNSCC, head and neck, EMT, TCGA, immunology, biomarker

## Abstract

MicroRNAs and their role in cancer have been extensively studied for the past decade. Here, we analyzed the biological role and diagnostic potential of *miR-154-5p* and *miR-154-3p* in head and neck squamous cell carcinoma (HNSCC). miRNA expression analyses were performed using The Cancer Genome Atlas (TCGA) data accessed from cBioPortal, UALCAN, Santa Cruz University, and Gene Expression Omnibus (GEO). The expression data were correlated with clinicopathological parameters. The functional enrichment was assessed with Gene Set Enrichment Analysis (GSEA). The immunological profiles were assessed using the ESTIMATE tool and RNAseq data from TCGA. All statistical analyses were performed with GraphPad Prism and Statistica. The study showed that both *miR-154-5p* and *miR-154-3p* were downregulated in the HNSCC samples and their expression levels correlated with tumor localization, overall survival, cancer stage, tumor grade, and HPV p16 status. GSEA indicated that individuals with the increased levels of *miR-154* had upregulated AKT-MTOR, CYCLIN D1, KRAS, EIF4E, RB, ATM, and EMT gene sets. Finally, the elevated *miR-154* expression correlated with better immune response. This study showed that *miR-154* is highly involved in HNSCC pathogenesis, invasion, and immune response. The implementation of *miR-154* as a biomarker may improve the effectiveness of HNSCC treatment.

## 1. Introduction

Head and neck squamous cell carcinomas (HNSCCs) account for about 90% of all cancers in the head and neck area and are among the most common neoplasms worldwide [1]. This group includes tumors of the upper part of the digestive and respiratory system, particularly the oral cavity, larynx, and pharynx [2]. HNSCC is typically diagnosed at the advanced stage, resulting in high morbidity and mortality [3]. It is mainly associated with exposure to tobacco, alcohol abuse, and human papillomavirus (HPV), particularly type 16, which plays a crucial role in HNSCC development [4,5]. HPV-positive HNSCC tumors are more frequently found in the oropharyngeal area, with a younger age of onset, and also show less molecular diversity and higher sensitivity to radiation or chemotherapy when compared to HPV-negative tumors [6]. Moreover, HPV-positive HNSCC patients have significantly longer overall survival (OS) [6]. However, the five-year survival rate in HNSCC is significantly lower compared with other malignancies such as breast, cervical, or colorectal cancer [7]. The high mortality rate is associated with a lack of diagnostic biomarkers, leading to failure in early diagnosis and insufficient effectiveness of therapeutic methods [7]. Molecular biomarkers are important tools to diagnose, assess the likely course of the disease, and predict response to the treatment. Additionally, they are necessary to implement personalized treatment [8,9]. Although many biomarkers seem to affect the diagnosis and prognosis of HNSCC patients, only a few of them are approved for clinical use [7,10].

MicroRNAs (miRNAs) are single-stranded ncRNA molecules with 21–23 nucleotides in length. They are involved in the post-transcriptional regulation of gene expression through translational inhibition or even destabilization of the targeted messenger RNA (mRNA) [11]. They play a pivotal role in maintaining tissue homeostasis by regulating processes such as cell proliferation, differentiation, or apoptosis [12]. miRNAs can act as tumor suppressors or oncogenes. The discovery of their exact role is pivotal for understanding the molecular pathways involved in the development of HNSCC, both HPV-negative and HPV-positive [13,14,15,16]. miRNAs have been extensively studied as potential cancer biomarkers mainly due to their stability in a variety of biological materials, their specificity, and because they are easy to process and analyze with current protocols [17].

In this study, using data from The Cancer Genome Atlas (TCGA), we analyzed the expression and biological role of *miR-154* strands *5p* and *3p* and determined their potential utility as HNSCC biomarkers. It is known that the expression level of *miR-154* is down-regulated in melanoma [18], glioma [19], non-small-cell lung (NSCLC) [20], breast [21], bladder [22], gastric cancers [23], and laryngeal squamous cell carcinoma [24] and up-regulated in renal cell carcinoma [25]. Analysis of *miR-154* functions revealed that this miRNA acts as a regulator of proliferation, cell viability, invasion, and migration, apoptosis of cancer cells in vitro and regulates tumor growth in vivo [18,22,23,24,25,26]. Nevertheless, there is a lack of studies discussing how each *miR-154* strand is expressed and how they affect the molecular characteristics of the tissue or the immune profile of the patients. In recent years, many reports have emphasized the importance of analyzing both miRNA strands separately [27,28,29,30]. It has been proven that these molecular species can be co-expressed in different tissues and display different modulatory roles in tumorigenic processes independently and/or cooperatively [27,28,29,30]. Therefore, within this study, we performed such an analysis of *miR-154* for the first time. Our in silico investigation was based on a cohort of HNSCC patients and aimed to elucidate whether *miR-154* could be further considered for clinical applications. 

## 2. Materials and Methods

### 2.1. Data Collection

The TCGA expression data of *miR-154-3p*, *miR-154-5p*, and selected genes, along with clinical data, were downloaded from the cBioPortal developed at Memorial Sloan Kettering Cancer Center, USA (Head and Neck Squamous Cell Carcinoma, TCGA, Provisional, 530 samples dataset) [31], UALCAN databases (http://ualcan.path.uab.edu/index.html; level 3 TCGA RNA-seq data (accessed on 9 December 2021)) [32] and using USCS Xena Browser from the University of California, Santa Cruz databases, USA (TCGA Head and Neck Cancer; dataset ID: TCGA.HNSC.sampleMap/HiSeqV2_PANCAN (accessed on 9 December 2021); pan-cancer normalized log2(norm_count + 1); and dataset ID: TCGA.HNSC.sampleMap/HNSC_clinicalMatrix (accessed on 9 December 2021) [33]. For validation of the results, available datasets GSE31277 (*n* = 16) [34] and GSE144711 (*n* = 88) [35] from Gene Expression Omnibus (GEO) (accessed on 9 December 2021)were used.

Additional information about the tumors’ biological and genetic features was obtained from the supporting data presented by Thorsson et al. [36].

All the data are available online with unrestricted access and does not require the patients’ consent or other permissions. The use of the data does not violate the rights of any person or any institution.

### 2.2. Pathological and Clinical Analysis

The expression levels of *miR-154-3p* and *miR-154-5p* were analyzed depending on the clinicopathological parameters such as age (≤60 vs. >60; *n* = 250 and *n* = 269 for *-5p* and *n* = 247 and *n* = 264 for *-3p*), gender (women vs. men; *n* = 147 and *n* = 373 for *-5p* and *n* = 43 and *n* = 369 for *-3p*), documented alcohol history (yes vs. no; *n* = 337 and *n* = 173 for *-5p* and *n* = 331 and *n* = 171 for *-3p*), tobacco smoking history (yes vs. no/ex; *n* = 302 and *n* = 203 for *-5p* and *n* = 296 and *n* = 201 for *-3p*), cancer stage (I + II vs. III + IV; *n* = 125 and *n* = 382 for *-5p* and *n* = 123 and *n* = 376 for *-3p*), T-stage (T1 + T2 vs. T3 + T4; *n* = 189 and *n* = 316 for *-5p* and *n* = 189 and *n* = 308 for *-3p*), N-stage (N0 vs. N2 + N2 + N3; *n* = 252 and *n* = 248 for *-5p* and *n* = 245 and *n* = 246 for *-3p*), cancer grade (G1 + G2 vs. G3 + G4; *n* = 370 and *n* = 126 for *-5p* and *n* = 361 and *n* = 127 for *-3p*), HPV p16 marker (positive vs. negative; *n* = 38 and *n* = 65 for *-5p* and *n* = 36 and *n* = 65 for *-3p*), perineural invasion (positive vs. negative; *n* = 171 and *n* = 192 for *-5p* and *n* = 169 and *n* = 188 for *-3p*), and lymphoid neck dissection status (positive vs. negative; *n* = 416 and *n* = 102 for *-5p* and *n* = 407 and *n* = 102 for *-3p*) for all the HNSCC anatomical subsites available. Next, subgroups of high and low expression of *miR-154-5p* and *miR-154-3p* were selected using the average expression as a cutoff. Disease-free survival (DFS) (*n_high_* = 215 and *n_low_* = 204 for *-5p* and *n_high_* = 194 and *n_low_* = 218 for *-3p*) and OS (*n_high_* = 258 and *n_low_* = 259 for *-5p* and *n_high_* = 248 and *n_low_* = 261 for *-3p*) were assessed in these subgroups.

For the GSE31277 dataset from GEO, the expression levels of *miR-154-5p* and *miR-154-3p* were analyzed depending on sample type (cancers vs. surgical margin; *n* = 15 and *n* = 15 for both), T-stage (T1 + T2 vs. T3 + T4; *n* = 6 and *n* = 9 for both), N-stage (N0 vs. N2 + N2 + N3; *n* = 6 and *n* = 9 for both), and histological differentiation (well-differentiated vs. moderate; *n* = 10 and *n* = 5 for both). The second GEO dataset, GSE144711, was analyzed according to cancer stage (I + II vs. III + IV; *n* = 13 and *n* = 53 for both), T-stage (T1 + T2 vs. T3 + T4; *n* = 17 and *n* = 54 for both), N-stage (N0 vs. N2 + N2 + N3; *n* = 29 and *n* = 42 for both), and histological differentiation (well-differentiated vs. moderate vs. poor; *n* = 15, *n* = 55, and *n* = 13 for all three). 

### 2.3. Functional Enrichment Analysis and Prediction of Genes’ Function

Gene Set Enrichment Analysis (GSEA) software version 3.0 (http://www.gsea-msigdb.org/gsea/index.jsp (University of California, San Diego, USA, and Broad Institute, USA, accessed on 9 December 2021) was used for the analysis of functional enrichment [37]. The HNSCC patients were divided into two groups with high and low expression of *miR-154* using the average expression as a cutoff. The input file contained expression data for 20,530 genes and 512 patients. We used 1000 gene set permutations for the analysis and pathways (oncogenic signatures (C), hallmark gene sets (H), and collection from MSigDB) with nominal *p*-value ≤0.05 and FDR *q*-value ≤0.25 considered significant. 

### 2.4. Target Analysis

The genes negatively correlated with *miR-154* were verified as potential targets using data from the Encori database (StarBase 2.0, Sun Yat-sen University, China, accessed on 9 December 2021) comparing information from di9 December 2021erent prediction tools: PITA (Segal Lab of Computational Biology, Israel, accessed on 9 December 2021), RNA22 (Thomas Jefferson University, USA, accessed on 9 December 2021) [38], miRmap (https://mirmap.ezlab.org/app/ (Swiss Institute of Bioinformatics, Switzerland, accessed on 9 December 2021)) [39], microT-CDS (http://diana.imis.athena-innovation.gr/DianaTools/index.php?r=microT_CDS/index (University of Thessaly, Grece, accessed on 9 December 2021)) [40,41], miRDB (http://www.mirdb.org (University School of Medicine, St Louis, MO, USA, accessed on 9 December 2021)) [42,43], PicTar (https://pictar.mdc-berlin.de (Center for Comparative Functional Genomics and the Max Delbruck Centrum, Germany, accessed on 9 December 2021)), and TargetScanHuman v7.2 (http://www.targetscan.org/vert_72/ (Bioinformatics and Research Computing (Whitehead Institute), USA, accessed on 9 December 2021)) [44]. Next, the correlation between expression levels of *miR-154* and genes was calculated as described in Section 2.6. The interactions between genes predicted as targets for *miR-154* were analyzed using the GeneMANIA prediction tool (http://genemania.org (University of Toronto, Canada, accessed on 9 December 2021)) [45]. DFS and OS of the HNSCC patients depending on the expression level of *miR-154* targets were analyzed using data from the gene expression profiling interactive analysis (GEPIA 2) portal (http://gepia2.cancer-pku.cn/#index (Peking University, China, accessed on 9 December 2021)). The log-rank test with a hazard ratio (HR) and 95% CI (confidence interval) was applied, and subgroups of the patients divided based on the quartile of the expression level of a specific gene and for the panel of all the indicated *miR-154-5p* targets were compared [46].

### 2.5. Immune Profile Analysis

Analysis of the ESTIMATE, immune and stromal scores (Estimation of Stromal and Immune cells in Malignant Tumor tissues using Expression data) required downloading a specific dataset from https://bioinformatics.mdanderson.org/estimate/disease.html (MD Anderson Cancer Center, USA, accessed on 9 December 2021) [47]. Then, those scores were used to define the infiltration of immune cells into tumor tissues and infer tumor purity. Subpopulations of specific immune cells were assessed using supporting data presented by Thorsson et al. and compared between subgroups of the patients with high and low expression of *miR-154-5p*, as well as of *miR-154-3p* [36]. Individuals were assigned into groups based on the mean value of the studied miRNA expression level.

Populations of specific immune cells, lymphocytes (T and B cells with their subpopulations), neutrophils, eosinophils, mast cells, dendritic cells, and macrophages (M0, M1, and M2), as well as T cell receptor (TCR) and B cell receptor (BCR) parameters, TCGA immune subtypes (C1–C4 and C6), along with other immune features such as IFN gamma and transforming growth factor beta (TGF beta) response, macrophage regulation, lymphocyte infiltration, TIL regional fraction, intratumor heterogeneity, proliferation, wound healing, homologous recombination defects, SNV and indel neoantigens, silent and non-silent mutation rates, aneuploidy, and cancer/testis antigen score (CTA score) were determined by conducting analyses of the supporting data presented by Thorsson et al. [36]. The data downloaded for this study can be explored and visualized through the interactive portal CRI iAtlas (www.cri-iatlas.org (Institute for Systems Biology, USA, accessed on 9 December 2021)) [48]. The analysis was performed as described in Section 2.6 “Statistical Analysis”.

### 2.6. Statistical Analysis

All the statistical analyses were performed using GraphPad Prism 8 (GraphPad, San Diego, CA, USA) and Statistica v13.1 (Dell Software, Round Rock, TX, USA). The Shapiro–Wilk normality test, *t*-test, or Mann-Whitney U test was used for *miR-154-5p* and *miR-154-3p* expression levels (depending on the clinical parameters). The level of *miR-154-5p* and *miR-154-3p* (depending on the National Institute of Health classification of cancer location, as well as the TCGA molecular subtype) was determined using one-way ANOVA with Tukey’s multiple comparisons test or the Kruskal-Wallis test with Dunn’s multiple comparisons test. A negative correlation between *miR-154-5p* and *miR-154-3p* expression levels, as well as protein-coding genes, were analyzed in Statistica (correlation matrix) or GraphPad Prism 8 with the Pearson or Spearman’s correlation tests depending on the data distribution. All the TCGA data are displayed as the means with the standard error of the mean (SEM). For DSF and OS analyses, the log-rank (Mantel-Cox) and Gehan-Breslow-Wilcoxon tests were used, and the hazard ratio (Mantel-Haenszel; HR) and the 95% confidence interval (CI) of the ratio were calculated. Graphs presenting boxes use 5–95 percentile whiskers, and column bars show the means with 95% CI. In all the analyses, *p* < 0.05 was used to determine statistical significance.

### 2.7. Availability of Data and Materials

The datasets used and/or analyzed during this study are available from the corresponding author on reasonable request. Raw data are available on the cBioPortal, in the UALCAN and the University of California, Santa Cruz databases, and the published Supplementary Materials.

## 3. Results

*miR-154-5p* and *miR-154-3p* were downregulated in the cancer samples when compared to the cancer-free samples, in pharyngeal squamous cell carcinomas and in the atypical molecular subtype of HNSCC.

**Figure 1:**
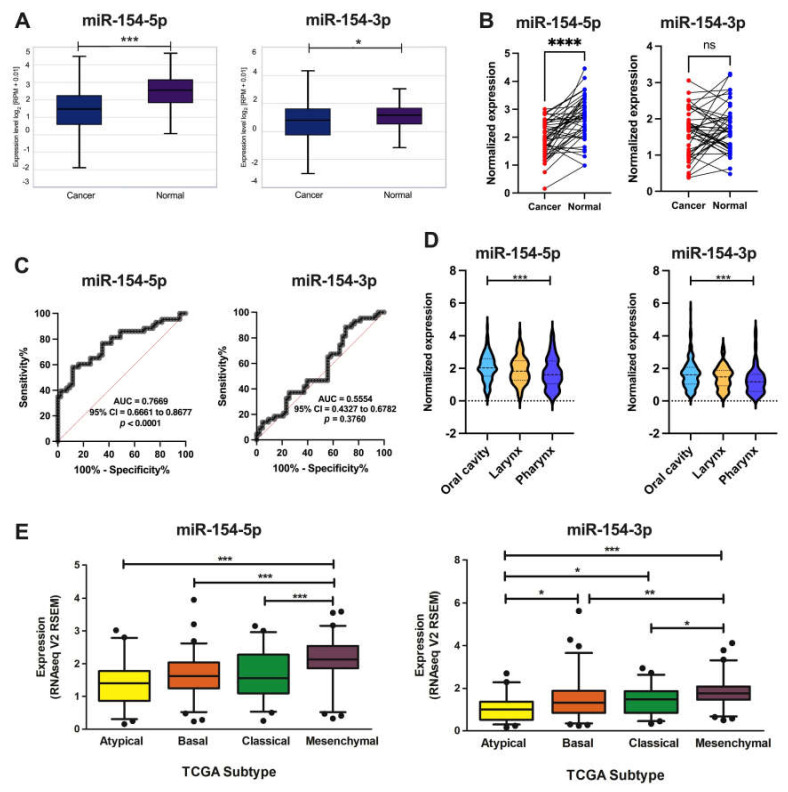
The expression level of *miR-154-5p* and *miR-154-3p* in the TCGA patients: (**A**) in the whole group of the HNSCC cancer (*n* = 497) and normal (*n* = 44) samples (unpaired analysis, graphs from the Encori database, modified) and (**B**) in the paired cancer and adjacent normal samples (*n* = 43); (**C**) ROC analysis of the paired cancer and adjacent normal samples (*n* = 43); (**D**) expression of *miR-154-5p* and *miR-154-3p* depending on HNSCC localization in the oral cavity (*n* = 323 for *miR-154-5p*; *n* = 316 for *miR-154-3p*), larynx (*n* = 117 for *miR-154-5p*; *n* = 116 for *miR-154-3p*), and pharynx (*n* = 80 for *miR-154-5p*; *n* = 80 for *miR-154-3p*), and (**E**) expression of *miR-154-5p* and *miR-154-3p* depending on the TCGA molecular subtype of HNSCC: atypical (*n* = 55), basal (*n* = 71 for *miR-154-5p*; *n* = 68 for *miR-154-3p*), classical (*n* = 44), or mesenchymal (*n* = 64 for *miR-154-5p*; *n* = 65 for *miR-154-3p*). The graphs show the mean expression and boxes and whiskers with the minimum to maximum values, or the 5–95 percentile; AUC—area under the ROC (receiver operating characteristic) curve, CI—confidence interval; *p* < 0.05 considered as significant, ns—not significant, * *p* < 0.05; ** *p* ≤ 0.01, *** *p* ≤ 0.001, **** *p* ≤ 0.0001.

According to the Encori database (StarBase 2.0), the expression of *miR-154-5p* was significantly downregulated in the HNSCC samples (*n* = 497) compared to the normal tissue (*n* = 44), with a fold change of 0.52 (*p* = 3.6 × 10^−6^). The expression level of *miR-154-3p* in the normal (*n* =  44) and HNSCC tissues (*n* =  497) was significantly downregulated, with a fold change of 0.97 (*p* = 0.024) (Figure 1A). Subsequently, the changes were examined based on paired cancer and adjacent normal samples analysis (*n* = 43), and *miR-154-5p* expression levels were significantly different between the normal and HNSCC tissues (1.940 ± 0.09783 vs. 2.676 ± 0.1207, *p* < 0.0001) while no differences were observed for *miR-154-3p* (1.596 ± 0.09891 vs. 1.736 ± 0.09794, *p* = 0.2192) (Figure 1B). Next, ROC analysis of the paired cancer and adjacent normal samples (*n* = 43) was performed, and in the case of *miR-154-5p*, high ability to distinguish between the cancer and normal samples was determined (*AUC* = 0.7669, *95% CI* = 0.6661–0.8677, *p* < 0.0001), in contrast to *miR-154-3p* which did not display this feature (*AUC* = 0.5554, *95% CI* = 0.4327–0.6782, *p* = 0.3760) (Figure 1C).

Based on the National Institutes of Health (NIH) classification, all the samples were divided into three main cancer groups according to subsite: (i) oral cavity (*n* = 323 for *miR-154-5p*; *n* = 316 for *miR-154-3p*), (ii) larynx (*n* = 117 for *miR-154-5p*; *n* = 116 for *miR-154-3p*), and (iii) pharynx (*n* = 80 for *miR-154-5p*; *n* = 80 for *miR-154-3p*). Significantly higher expression of *miR-154-5p* and *miR-154-3p* was observed for tumors located in the oral cavity when compared with the pharynx (2.058 ± 0.04528 vs. 1.729 ± 0.1067, *p* = 0.0051 and 1.644 ± 0.04764 vs. 1.272 ± 0.09289, *p* = 0.0008, respectively). No significant differences (*p* > 0.05) were determined between other subsites (Figure 1D). Finally, expression levels of *miR-154-5p* and *miR-154-3p* were examined depending on the molecular subtype of HNSCC. The highest levels of *miR-154-5p* were detected in the mesenchymal subtype (2.123 ± 0.08424, *p* < 0.001; *n* = 64) in comparison to the atypical (1.390 ± 0.08961; *n* = 55), basal (1.627 ± 0.08081; *n* = 71), or classical subtypes (1.634 ± 0.1091; *n* = 44), and no differences (*p* > 0.05) were determined between the atypical vs. basal and classical subtypes as well as between the basal and classical subtypes. Similarly, in the case of *miR-154-3p*, the highest levels were observed in the mesenchymal subtype (1.811 ± 0.08717, *p* < 0.0001; *n* = 64) in comparison to the atypical (1.047 ± 0.08116; *n* = 55), basal (1.486 ± 0.1176; *n* = 68), or classical subtypes (1.414 ± 0.09748; *n* = 44), and no differences were determined between the basal vs. classical subtypes (*p* > 0.05) (Figure 1E).

### 3.1. miR-154-5p and miR-154-3p Levels Differ Depending on the Clinicopathological Parameters

The expression levels of *miR-154-5p* and *miR-154-3p* were analyzed in the previously determined subgroups depending on the available clinicopathological parameters for all the HNSCC samples. Significant differences in the expression levels for *miR-154-5p* were observed in the N0 vs. N1 + N2 + N3 cancer N-stages (2.071 ± 0.8426 vs. 1.866 ± 0.8409, *p* = 0.0066), I + II vs. III + IV cancer stages (2.145 ± 0.8002 vs. 1.913 ± 0.8548, *p* = 0.0077), G1 + G2 vs. G3 + G4 cancer grades (1.913 ± 0.8247 vs. 2.182 ± 0.8299, *p* = 0.0017), and in the negative HPV p16/ish samples in comparison to the positive ones (1.492 ± 0.9784 vs. 1.943 ± 0.7552, *p* = 0.0102). The other analyzed parameters did not differ between the studied groups for *miR-154-5p*. Significant differences between the *miR-154-3p* expression levels were detected only between the N0 vs. N1 + N2 + N3 cancer N-stages (1.623 ± 0.8086 vs. 1.455 ± 0.7938, *p* = 0.0208) and in the negative HPV p16/ish samples in comparison to the positive ones (1.208 ± 0.7566 vs. 1.534 ± 0.6708, *p* = 0.0277); the other analyzed parameters did not vary between the studied groups. All the data are presented in Table 1.

**Figure 2:**
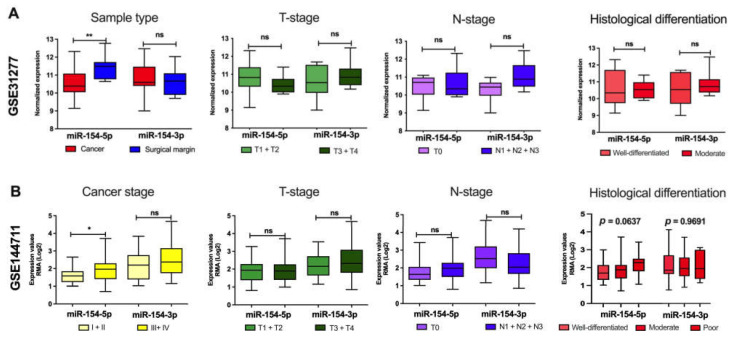
Expression levels of *miR-154-5p* and *miR-154-3p* in the HNSCC patients from GEO depending on sample type, stages of cancer, and histological differentiation based on datasets (**A**) GSE31277 (*n* = 16) and (**B**) GSE144711 (*n* = 88). The graphs show the mean expression and boxes and whiskers with the minimum to maximum values; *p* < 0.05 considered as significant, ns—not significant, * *p* < 0.05; ** *p* ≤ 0.01.

Next, the results based on the TCGA data were tested using datasets GSE31277 and GSE144711 from the GEO database, and the expression levels of *miR-154-5p* and *miR-154-3p* depending on sample type, stages of cancer, and histological differentiation were assessed. In the case of GSE31277, significantly lower expression levels of *miR-154-5p* in the cancer samples in comparison to the surgical margin were observed (10.58 ± 0.1930 vs. 11.39 ± 0.1546, *p* = 0.0028), and no differences were observed for *miR-154-3p* (10.79 ± 0.2095 vs. 10.54 ± 0.1884, *p* = 0.3840). Moreover, no differences in the expression levels between the T- and N-stages as well as histological differentiation for both *miR-154-5p* (10.81 ± 0.4150 vs. 10.42 ± 0.1705, *p* = 0.2721; 10.48 ± 0.2890 vs. 10.64 ± 0.2696, *p* = 0.9546; and 10.54 ± 0.1617 vs. 10.64 ± 0.5214, *p* = 0.9530, respectively) and *miR-154-3p* (10.59 ± 0.3918 vs. 10.93 ± 0.2416, *p* = 0.6663; 10.29 ± 0.2760 vs. 11.12 ± 0.2484, *p* = 0.0933; and 10.88 ± 0.2226 vs. 10.62 ± 0.4764, *p* = 0.8825, respectively) were detected (Figure 2A). Next, analysis of the GSE144711 dataset revealed that *miR-154-5p* was upregulated in cancer with higher (III + IV) stages in comparison to the samples with I + II stages (1.603 ± 0.1322 vs. 1.951 ± 0.09924, *p* = 0.0028); however, those differences were not observed for *miR-154-3p* (2.161 ± 0.2289 vs. 2.447 ± 0.1106, *p* = 0.2756). Additionally, no differences were observed for the T- and N-stages for both *miR-154-5p* (1.857 ± 0.1534 vs. 1.925 ± 0.08823, *p* = 0.2721, and 1.764 ± 0.1166 vs. 2.003 ± 0.09912, *p* = 0.9546, respectively) and *miR-154-3p* (2.203 ± 0.1798 vs. 2.384 ± 0.1105, *p* = 0.6663, and 2.530 ± 0.1483 vs. 2.227 ± 0.1199, *p* = 0.9530, respectively) and histological differentiation (1.781 ± 0.1419 vs. 1.833 ± 0.08736 vs. 2.229 ± 0.1846, *p* = 0.0637, and 2.356 ± 0.2588 vs. 2.306 ± 0.09991 vs. 2.350 ± 0.2417, *p* = 0.0933, respectively) (Figure 2B).

### 3.2. Patients with Low Expression of miR-154-3p Had a Significantly Extended Overall Survival

**Figure 3:**
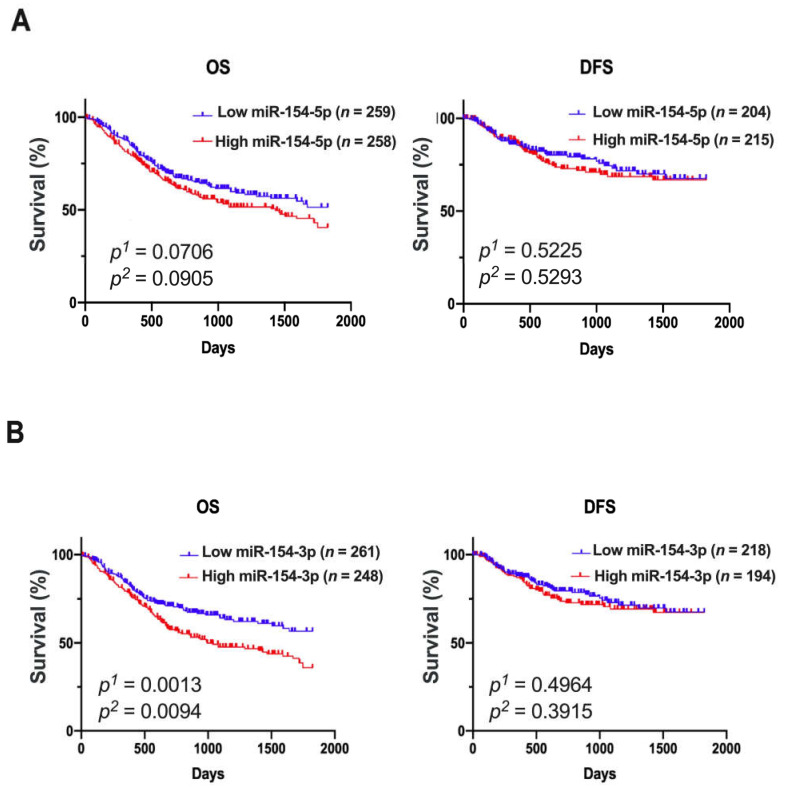
DFS and OS of the HNSCC patients depending on the *miR-154-5p* (**A**) or *miR-154-3p* (**B**) expression levels; low and high subgroups of patients divided based on the mean expression; *p*^1^—log-rank (Mantel-Cox) test, *p*^2^—Gehan–Breslow-Wilcoxon test; *p* < 0.05 considered as significant.

The HNSCC patients were divided into low and high *miR-154-3p* and *miR-154-5p* expression groups using the mean expression as a cutoff, and then the 5-year OS and DFS were assessed. Slight changes in the OS time between low and high *miR-154-5p* expression groups were observed (*p* = 0.0706 and *p* = 0.0905, respectively), and no differences (*p* > 0.05) for the DFS were seen (Figure 3A). However, a significantly longer 5-year OS was discovered in the patients with low *miR-154-3p* expression levels in comparison to the high expression group (*p* = 0.0013 and *p* = 0.0094, respectively). In the case of DFS, no difference between the patients with low and high expression levels of *miR-154-3p* was determined (Figure 3B).

### 3.3. Higher Expression of miR-154-5p and miR-154-3p Was Associated with the Upregulation of Oncogenic Pathways in the HNSCC Patients

**Figure 4:**
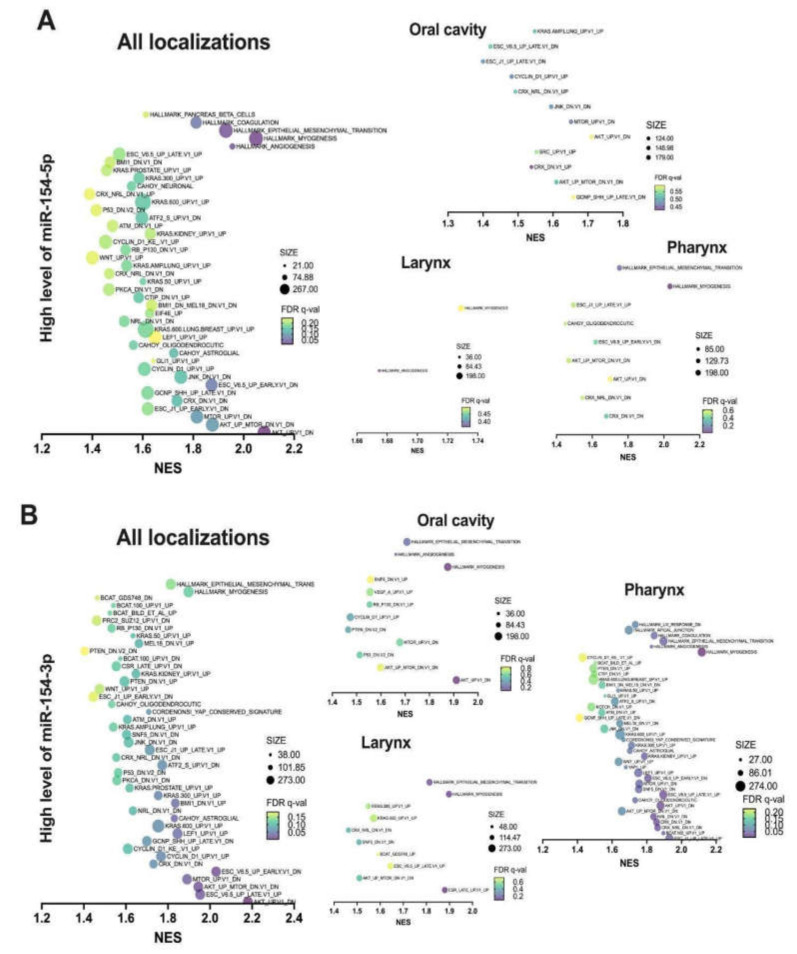
GSEA of the MSigDB gene sets enriched in the HNSCC patients with a higher expression level of *miR-154-5p* (**A**) and a higher expression level of *miR-154-3p* (**B**) in all the anatomical subsites together as well as distinguished specifically into the oral cavity, larynx, or pharynx. Only the gene sets with nominal *p* < 0.05 were presented; NES—normalized enrichment score, FDR *q*-value—false discovery rate, SIZE—number of enriched genes in a specified process.

The biological influence of high and low expression levels of *miR-154-5p* and *miR-154-3p* was analyzed using GESA. In the case of all the HNSCC subsites, significant enrichment scores (*p* < 0.05 and FDR *q*-value <0.25) were observed for 36 gene sets from oncogenic signatures and five gene sets from cancer hallmark signatures for the patients with higher levels of *miR-154-5p* compared to the ones with lower expression of this miRNA. Similarly, for high levels of *miR-154-3p*, significant enrichment in 41 gene sets from oncogenic signatures and two gene sets from cancer hallmark signatures were detected. In both cases, those genes were implicated in AKT-MTOR, CYCLIN D1, KRAS, EIF4E, RB, as well as ATM pathways. Moreover, patients with higher levels of both strands displayed gene expression signatures characteristic of cancer stem-like cells, with features including genes downregulated during early stages of differentiation or connected with the WNT pathway. The above was supported by the enrichment of 194 genes associated with the Epithelial-to-Mesenchymal (EMT) process (NES = 1.930 for *miR-154-5p* and NES = 1.813 for *miR-154-3p*, respectively). It should be noted that gene enrichment analysis depending on the cancer subsite (oral cavity, larynx, or pharynx) also suggests a strong association of higher levels of both *miR-154* strands with an aggressive phenotype of HNSCC. All the data are presented in Figure 4A,B and in Supplementary Table S1.

**Figure 5:**
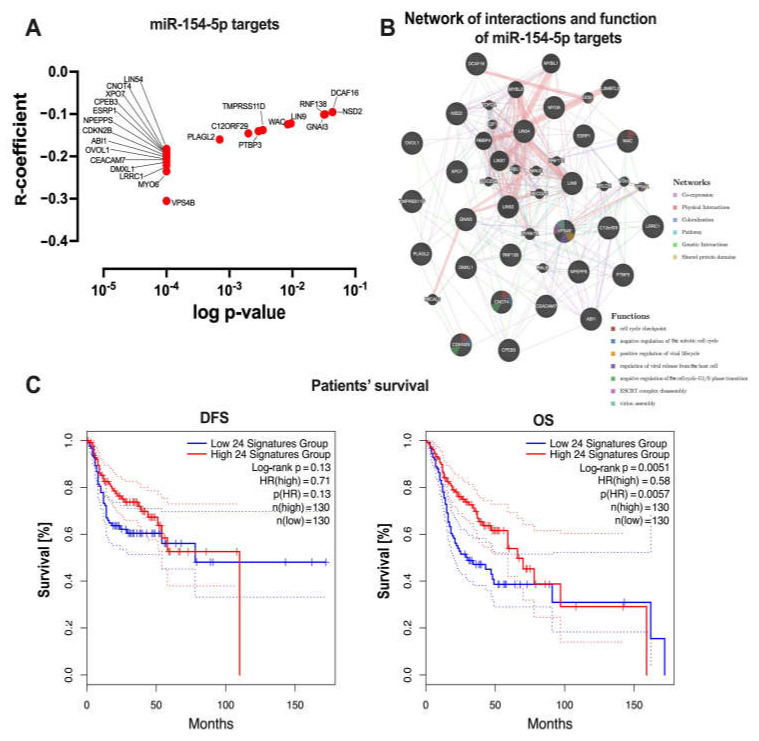
*miR-154-5p* targets. (**A**) correlation between *miR-154-5p* and the 24 genes selected based on the miRNA target prediction databases; only the significant (*p* < 0.05) genes were indicated. (**B**) Network of interactions and functions between the *miR-154-5p* targets selected using the GeneMANIA tool. (**C**) DFS and OS of the HNSCC patients depending on the expression level of the *miR-154-5p* targets. Data from the gene expression profiling interactive analysis (GEPIA 2) portal; curves (darker lines) with 95% CI (lighter lines); high and low expression level subgroups of patients divided based on the quartile of expression level; *n*—number of cases in each subgroup; *p*—log-rank test, HR—hazard ratio; CI—confidence interval; *p* < 0.05 considered as significant.

Next, gene sets from the processes selected by means of GSEA were screened for potential targets for *miR-154-5p* using miRNA target prediction databases, and the expression correlation between *miR-154-5p* and the genes was calculated. Based on this approach, 24 genes were selected: VPS4B, MYO6, LRRC1, DMXL1, CEACAM7, OVOL1, ABI1, CDKN2B, NPEPPS, ESRP1, CPEB3, XPO7, LIN54, CNOT4, PLAGL2, C12ORF29, PTBP3, TMPRSS11D, WAC, LIN9, RNF138, GNAI3, DCAF16, and NSD2 (with *R*-coefficient from −0.09532 to −0.3055 and *p* < 0.05) (Figure 5A). Using the GeneMANIA tool, the network of interactions and functions between the *miR-154-5p* targets was indicated. It was observed that those genes were co-expressed (61.19% of genes), had physical interactions (24.85% of genes), shared the same colocalization (7.83% of genes), and were from the same pathway (5.28% of genes). Moreover, these 24 genes are involved in the endosomal sorting complex required for transport (ESCRT), cell cycle, and viral replication (Figure 5B). Finally, the association of the *miR-154-5p* targets with the HNSCC patients’ survival was determined. In the case of higher expression of *CEACAM7* and *PLAGL2*, a significantly longer DFS was observed (*HR* = 0.58, *p* = 0.022; *HR* = *p* = 0.036). Surprisingly, only for higher expression levels of CPEB3, longer OS time was observed (*HR* = 0.56, *p* = 0.0045). However, when the expression levels of all the 24 genes were compiled, a strong association between higher expression levels and longer OS time for the HNSCC patients was observed (*HR* = 0.58, *p* = 0.0051). No differences for DFS time were observed (*HR* = 0.71, *p* = 0.13) (Figure 5C). The detailed data are presented in Supplementary Table S2.

### 3.4. Patients with Low Levels of Both miR-154 Mature Strands Have a More Favorable Immunological Profile

**Figure 6:**
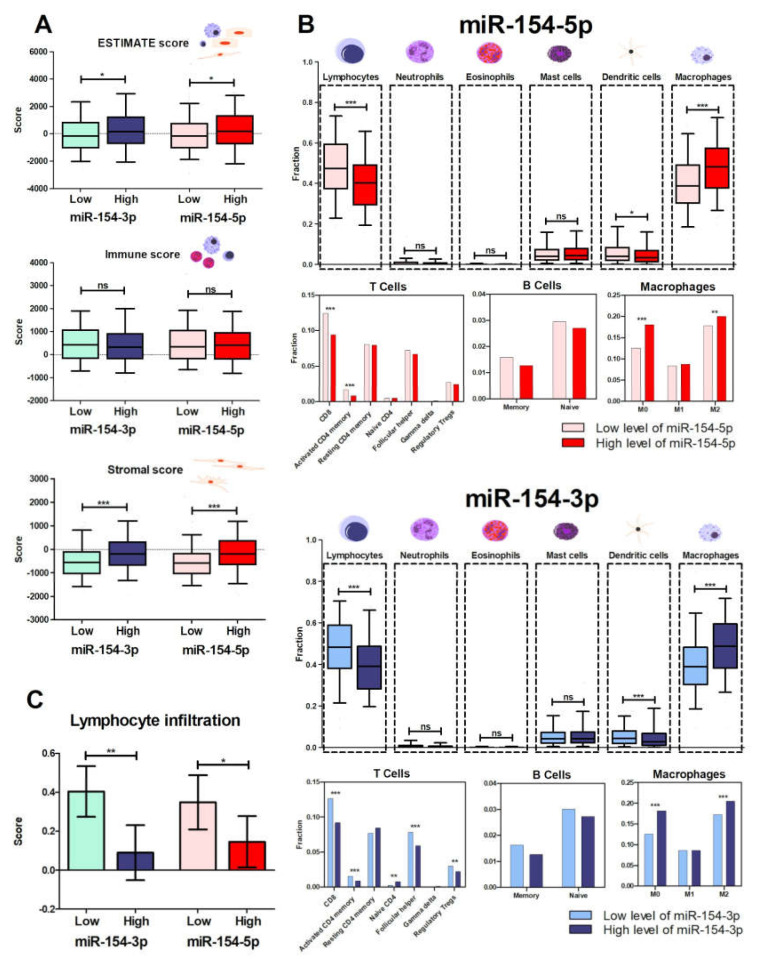
Immunological profile of the HNSCC patients depending on the expression level of *miR-154-5p* and *miR-154-3p*. Assessment of tumor purity and the presence of infiltrating stromal/immune cells in tumor tissues with the ESTIMATE, immune, and stromal scores (**A**); prediction of the lymphocyte infiltration signature score (**B**); estimation of differences of lymphocytes, neutrophils, eosinophils, mast cells, dendritic cells, and macrophages, as well as fractions of specific subpopulations of T cells, B cells, and macrophages (**C**); *t*-test, *p* < 0.05 considered as significant; ns—not significant, * *p* < 0.05, ** *p* ≤ 0.01, *** *p* ≤ 0.001.

The last step of this study was to assess the immune profile of the patients with high and low expression levels of *miR-154-5p* and *miR-154-3p*. The ESTIMATE analysis of the previously determined patient groups distinguished based on the mean miRNA expression values was conducted, and then the lymphocyte infiltration score and the distribution of the immune cell fractions among them were examined. Investigation of the ESTIMATE score, which determines the cellular composition (purity) of the tumor, showed statistically significant differences between individuals with high and low levels of *miR-154-5p* (*p* = 0.0112) as well as of *miR-154-3p* (*p* = 0.0373). Next, the immune score was analyzed, but no significant (*p* < 0.05) differences between the studied subgroups were observed. Subsequently, the stromal score, which indicates the presence of stroma in tumor tissue was assessed. It was indicated that the patients with low expression of both *miR-154* strands had a significantly decreased stromal score (*p* < 0.0001 and *p* < 0.0001, respectively). All the data are presented in Figure 6A.

Even though the immune score did not show any differences between the studied groups, the infiltration of lymphocytes varied. Individuals with low expression levels of both *miR-154-5p* and *miR-154-3p* showed a higher lymphocyte infiltration signature score compared to the high expression subgroups (*p* = 0.0377 and *p* = 0.0014, respectively) (Figure 6B).

Finally, to complete the immunological profile of the patients, the fractions of lymphocytes, neutrophils, eosinophils, mast cells, dendritic cells, and macrophages were investigated. The patients with decreased levels of *miR-154-3p* and *miR-154-5p* manifested with an increased number of lymphocytes (0.4007 ± 0.009664 vs. 0.4825 ± 0.009494, *p* < 0.0001, and 0.4075 ± 0.009063 vs. 0.4770 ± 0.009941, *p* < 0.0001, respectively), as well as of dendritic cells (0.06019 ± 0.003871 vs. 0.04947 ± 0.003394, *p* = 0.0188, and 0.05744 ± 0.003241 vs. 0.05125 ± 0.004142, *p* = 0.0009, respectively). These individuals also had a less abundant fraction of macrophages in comparison to the high expression subgroups (0.3993 ± 0.009099 vs. 0.4799 ± 0.008778, *p* < 0.0001, and 0.3973 ± 0.008668 vs. 0.4856 ± 0.009344, *p* < 0.0001, respectively). Interestingly, analysis of T cell types showed precisely that the patients with decreased expression of both *miR-154* strands were associated with an elevated number of CD8 cells (0.1244 ± 0.005575 vs. 0.09381 ± 0.004708, *p* < 0.0001, and 0.1265 ± 0.005365 vs. 0.09202 ± 0.004946, *p* < 0.0001, respectively) and activated CD4 memory T cells (0.01663 ± 0.001796 vs. 0.008100 ± 0.001094, *p* = 0.0002, and 0.01528 ± 0.001663 vs. 0.008664 ± 0.001272, *p* < 0.0001). Additionally, the low level of *miR-154-3p* was associated with an increased fraction of follicular helper (0.07836 ± 0.003174 vs. 0.05919 ± 0.002985, *p* < 0.0001) and regulatory T cells (0.02939 ± 0.001925 vs. 0.02206 ± 0.001910, *p* = 0.0019), along with the decreased number of CD4 naive cells (0.002255 ± 0.0006719 vs. 0.007444 ± 0.001656, *p* = 0.0055). Analysis of the different types of macrophages determined that the low *miR-154-5p* and *miR-154-3p* expression groups displayed a decreased fraction of M0 (0.1251 ± 0.008437 vs. 0.1806 ± 0.009722, *p* < 0.0001, and 0.1257 ± 0.008518 vs. 0.1819 ± 0.009964, *p* < 0.0001, respectively), as well as of M2 cells (0.1776 ± 0.005108 vs. 0.2002 ± 0.005761, *p* = 0.0035, and 0.1728 ± 0.005058 vs. 0.2048 ± 0.005938, *p* < 0.0001, respectively). All of the above data are presented in Figure 6C.

**Figure 7:**
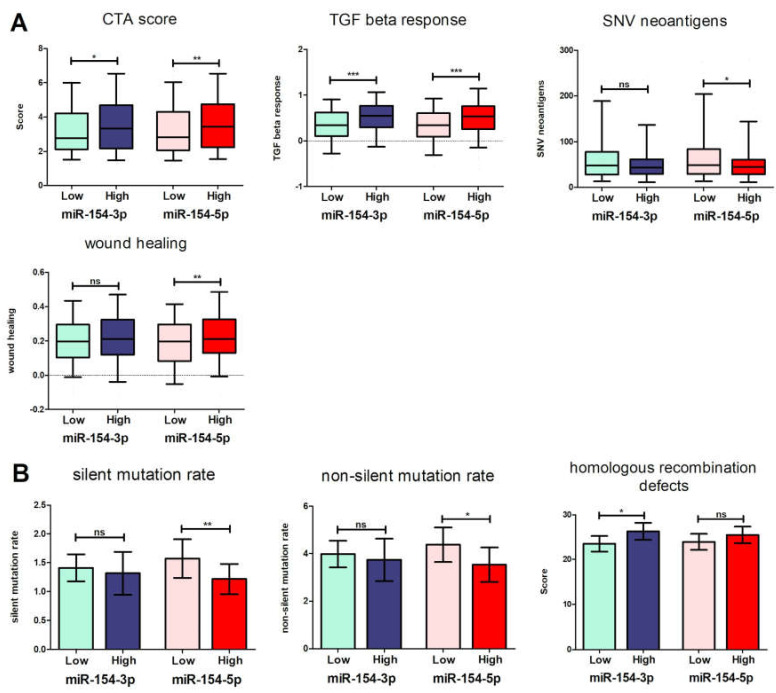
Immunological characteristics including the estimated CTA score, TGF beta response, SNV neoantigens, and wound healing (**A**) and the level of silent and non-silent mutations, as well as homologous recombination defects (**B**) depending on high and low expression of *miR-154-5p* and *miR-154-3p* in the HNSCC patients; *t*-test, *p* < 0.05 considered as significant; ns—not significant, * *p* < 0.05, ** *p* ≤ 0.01, *** *p* ≤ 0.001.

Considering the potential strong effect of the studied miRNA on immune cells, we decided to conduct analyses on the other data provided by Thorsson et al. concerning immune features [36]. It was found that the individuals with low expression levels of *miR-154-3p* and *miR-154-5p* manifest with a decreased CTA score (23.47 ± 0.8757 vs. 26.22 ± 0.9680, *p* = 0.0250, and 23.89 ± 0.9088 vs. 25.45 ± 0.9260, *p* = 0.0096, respectively) and weakened TGF beta response (0.3419 ± 0.02382 vs. 0.5294 ± 0.02439, *p* < 0.0001, and 0.3399 ± 0.02426 vs. 0.5129 ± 0.02402, *p* < 0.0001, respectively). Additionally, the group of patients with downregulated *miR-154-5p* had an elevated number of SNV neoantigens (74.77 ± 6.190 vs. 61.12 ± 7.012, *p* = 0.0358), along with worse wound healing (0.1883 ± 0.009551 vs. 0.2273 ± 0.009850, *p* = 0.0047) (Figure 7A).

Low levels of *miR-154-5p* were associated with elevated silent (1.570 ± 0.1701 vs. 1.216 ± 0.1324, *p* = 0.0067), and non-silent mutation rates (4.368 ± 0.3700 vs. 3.521 ± 0.3681, *p* = 0.0172). The patients with high *miR-154-3p* levels had an increased homologous recombination defect score in comparison to the low expression level group (23.47 ± 0.8757 vs. 26.22 ± 0.9680, *p* = 0.0243) (Figure 7B).

**Figure 8:**
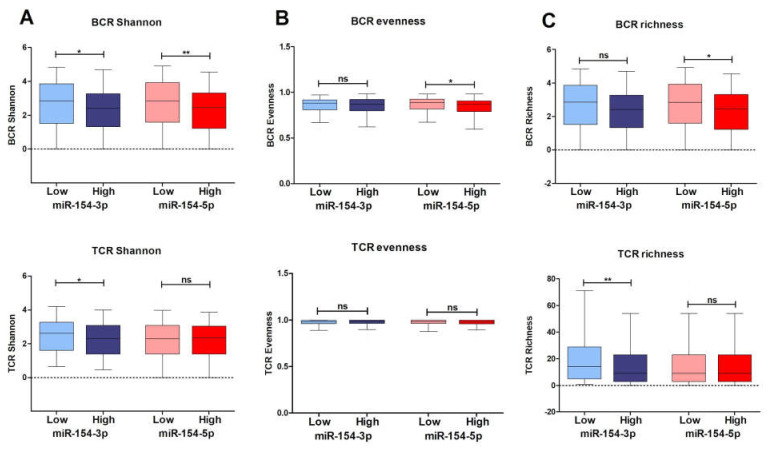
Assessment of the BCR and TCR Shannon (**A**), evenness (**B**), and richness (**C**) parameters depending on the expression level of *miR-154-5p* and *miR-154-3p* in the HNSCC patients; *t*-test, ns—not significant, *p* < 0.05 considered as significant; * *p* < 0.05, ** *p* ≤ 0.01.

Analysis of the TCR and BCR parameters revealed statistically significant differences between the BCR Shannon entropy scores in the low and high expression groups of *miR-154-3p* (2.662 ± 0.1064 vs. 2.321 ± 0.1092, *p* = 0.0191) and *miR-154-5p* (2.711 ± 0.1086 vs. 2.281 ± 0.1070, *p* = 0.0071) (Figure 8A). It was also found that the individuals with a low expression level of *miR-154-3p* manifested with an increased TCR Shannon entropy value (2.500 ± 0.07305 vs. 2.259 ± 0.08075, *p* = 0.0261), (Figure 8B) and richness scores (22.03 ± 1.625 vs. 17.57 ± 1.621, *p* = 0.0063) (Figure 8C) in comparison to the opposite group of patients. Interestingly, high levels of *miR-154-5p* were associated with decreased BCR evenness value (0.8393 ± 0.009476 vs. 0.8642 ± 0.007099, *p* = 0.0361) (Figure 8B) and richness scores (67.57 ± 6.809 vs. 44.97 ± 5.597, *p* = 0.0207) (Figure 8C).

## 4. Discussion

Head and neck cancers are a group of neoplasms with a strong ability to metastasize to local lymph nodes, which in consequence leads to a high mortality rate [1,49]. Moreover, the standard treatment based on radiotherapy, chemotherapy, as well as immunotherapy does not bring satisfactory results and needs to be improved [50,51,52]. Personalization of therapy requires the use of biomarkers, which could help to diagnose and predict the patient outcome. The molecular biomarkers seem to be the best option because they may describe molecular states of the cell and dictate treatment strategies [53,54,55]. Among the widely studied types of molecules, which could become valuable oncology biomarkers, are RNAs. It was demonstrated that both protein-coding and non-coding RNAs such as miRNAs or long non-coding RNAs (lncRNAs) are dysregulated in HNSCC and are potentially useful in clinical practice [56,57,58,59,60,61].

In this study, we used data from TCGA to analyze the expression and the biological role of *miR-154-5p* and *-3p* strands, as well as their usefulness as biomarkers in HNSCCs.

Our first important observation was that *miR-154-5p* and *miR-154-3p* are downregulated in HNSCC patients, and *miR-154-5p* specifically distinguishes cancer-free from cancer tissue. GEO datasets were used to validate the results obtained with TCGA data. Our results corroborate the previous studies regarding laryngeal squamous cell carcinoma, showing that the expression level of *miR-154* in tumors is decreased in comparison to the healthy tissue [18,19,20,21,22,23,24].

It was observed that the expression levels of *miR-154* are significantly downregulated in oral squamous cell carcinoma (OSCC) tissues in comparison to tumor-free surgical margins [34].

Moreover, we indicated that neoplasms situated in the pharynx had the lowest expression levels of *miR-154*; however, the difference was statistically significant only when compared to the oral cavity. Niu et al. analyzed the expression of *miR-154* and demonstrated its downregulation in laryngeal cancers, although the study focused only on the Chinese population [24]. In fact, extensive research concerning different HNSCC subsites is lacking. Our study analyzed TCGA data obtained from samples collected predominantly from Caucasians, and not Hispanic or Latino populations, but the results were confirmed using a GEO dataset generated from individuals from South America, improving the diversity and relevance of the findings.

We found that low expression of *miR-154-5p* and *miR-154-3p* was significantly associated with a higher N-stage. Decreased *miR-154-5p* was also linked with the III + IV cancer stages. Surprisingly, our results obtained using the GEO database were opposite; the expression of *miR-154-5p* was higher in stages III + IV. In agreement with TCGA dataset results, other studies showed that low levels of *miR-154* correlate with more advanced disease (higher grade and/or cancer stages) [24,62,63] and could reflect different study populations. We observed the trend of higher expression levels of *miR-154-5p* depending on the histological differentiation status, where expression of that miRNA was the highest in the poorly differentiated cancers (GEO) and within the mesenchymal subtype (TCGA). However, as it had been previously indicated in gliomas, low expression of *miR-154* was linked to the higher grade, large tumor size, and unfavorable Karnofsky score [19]. The same situation was described in the patients with bladder cancer, where *miR-154* downregulation was significantly associated with more advanced tumor staging, higher histologic grades, and lymph node metastasis [22]. On the other hand, in melanoma tissues, the increased level of *miR-154* correlated with higher stages of tumor development and ulceration [18].

In this study, there were no significant associations between *miR-154* expression and age or sex in the patients from our dataset, which stays in agreement with other studies on various cancers [18,19,22]. Furthermore, we observed that the patients with positive HPV p16 status displayed low levels of both *miR-154* strands. Vojtechova et al. indicated that two members of the *miR-154* family, *miR-323a-3p* and *miR-487a-3p*, were dysregulated in HPV-positive compared to HPV-negative tonsillar tumors [64]. *miR-154-5p* and *-3p* are downregulated in HPV-positive cervical cancer cell lines, but their roles were not examined by the authors [65]. This observation was confirmed by Zhao et al. using different cervical tissues and cell lines. Knockdown of *miR-154-5p* in HPV-16-positive SiHa cells caused increased cell proliferation, migration, along with the invasive ability, and its upregulation led to the reversed effect. They proved that *miR-154-5p* directly binds to the 3′UTR of CUL2, the core component of the E3 ubiquitin protein ligase complex, which, in turn, regulates the steady-state levels and stability of pRb [66]. It is worth mentioning that no reports of the direct role of *miR-154-5p* or *-3p* in HPV-positive HNSCC are available, and their diagnostic and biological role should be determined in the future. The ambiguity of associations between *miR-154* strands and various clinicopathological parameters corroborates the complex nature of this regulatory system. To draw precise conclusions, complex interaction networks should be considered, which we attempted with GSEA.

We investigated whether *miR-154-5p* and *miR-154-3p* could be used as prognostic biomarkers for the assessment of individuals with HNSCC. We observed that the patients with lower levels of *miR-154-3p* and *miR-154-5p* had better OS compared to the group with high expression. The results from a study by Lin et al. confirmed this finding in renal cell carcinoma; however, the expression level of this miRNA was upregulated in cancer tissues [25]. Niu et al. described the reverse correlation between *miR-154* and laryngeal cancer patient survival compared to our results [24]. Moreover, the same observation was proved for melanoma [18], glioma [19], and bladder cancer [22]. However, there is no available information about the influence of the *miR-154* expression level on the OS in NSCLC [20], breast cancer [21], and gastric cancer [23]. It should be emphasized that there are no other independent studies concerning *miR-154* in HNSCC than that presented by Niu et al. The authors were aware of the discrepancies between the results of their analyses, e.g., of the association of low *miR-154-3p* with a higher N-stage (tumor-suppressive character) and better OS (oncogenic role); however, these types of correlations rarely provide unequivocal results when based on a single miRNA. Moreover, it is well-known that the biology of neoplastic cells differs significantly from that of normal cells, and primary neutral miRNAs regulating tens or even hundreds of mRNA transcripts can be turned into pro- or antitumor molecules [67].

GSEA showed an association of higher levels of *miR-154-5p* and *miR-154-3p* with the enrichment of genes from oncogenic and cancer hallmark signature sets. The most dysregulated genes were related with the signaling pathways such as AKT-MTOR, CYCLIN D1, KRAS, EIF4E, RB, and ATM, which play an essential role in cancer cell proliferation, survival, and response to external stimuli. For example, E2F transcriptional activity is tightly regulated throughout the cell cycle via transcriptional and translational regulation, post-translational modifications, protein degradation, binding to cofactors, and subcellular localization. Furthermore, E2F participates in apoptosis and cell differentiation. Alterations in components of this pathway coincide with poor prognosis in cancers, emphasizing their importance for the clinical cancer phenotype. An intriguing addition to the understanding of E2F crosstalks was the finding that their activity could be regulated by miRNAs whose dysregulation has been implicated in malignancy. In turn, miRNAs themselves are targets of the E2F family proteins establishing negative feedback loops [68,69]. In breast cancer tissues, E2F5 was identified as a target of *miR-154* with the inversely correlated expression. Additionally, it has been reported that in breast cancer cell lines (MCF-7), overexpression of *miR-154* significantly inhibited cell proliferation, migration, and invasion, as well as increased cell arrest at the G0/G1 stage. These findings indicate that *miR-154* acts as a tumor suppressor targeting E2F5 in breast cancer [70]. In this study, we proved that genes from the E2F target dataset had a decreased expression in the group of patients with high *miR-154* levels. Moreover, those genes are potential targets of *miR-154-5p*. Thus, E2F could be recognized as a potential target of *miR-154*, and as a result of its degradation, there may be a reduction in the expression of these transcription factors’ target molecules.

The genes involved in the G2/M checkpoint play a crucial role in progression through the cell division cycle. The G2 checkpoint prevents cells from entering mitosis when DNA is damaged, providing an opportunity for repair and inhibition of damaged cells proliferation. When mammalian cells contain damaged DNA, the p53 tumor suppressor and the Rb family of transcriptional repressors work together to downregulate a broad number of genes that encode proteins required for the G2 and M checkpoints. Elimination of these essential cell cycle proteins helps to keep the cells arrested in G2 [71,72]. Many studies have confirmed that miRNAs are involved in cell cycle arrest in the G2/M phase. In renal tubular cells, hypoxia induces G2/M arrest, leading to renal fibrosis via the miR-493-STMN-1 pathway [73]. *miR-192* is responsible for the G2/M arrest in the proximal tubular epithelial cells after toxic injury by aristolochic acid [74]. On the other hand, evidence from endometriotic cells suggests that *miR-210-3p* attenuates the G2/M cell cycle checkpoint by inactivating the BRCA1 complex function in response to DNA damage under hypoxia via targeting the 3′ untranslated region of BARD1 mRNA [75].

We also observed enrichment in the TBK1 gene set supporting the data of Barbie et al. who studied the epithelial lung cancer cell lines upon overexpression of an oncogenic form of the KRAS gene and knockdown of the TBK1 gene by RNAi. In experiments on human cancer cell lines, the lack of TBK1 induced apoptosis which was specifically dependent on oncogenic KRAS expression. In these cells, TBK1 activated NF-kappaB antiapoptotic signals involving c-Rel and BCL-XL, which were essential for survival and provided mechanistic insights into this synthetic lethal interaction. These observations indicate that TBK1 and NF-kappaB signaling is fundamental in KRAS-mutated tumors and establishes a general approach for the rational identification of codependent pathways in cancer [76].

The most important finding from this study was that high *miR-154-5p* and *miR-154-3p* expression levels were positively correlated with the EMT pathway in the HNSCC patients. It should be emphasized that the EMT pathway was enriched in all the localizations of HNSCC tumors. It had been confirmed before that *miR-154* promoted EMT in prostate cancer cells. In several isogenic prostate cancers, the ARCaP and LNCaP lines that drive the EMT and bone metastasis in mice were significantly increased in populations with high *miR-154* levels. Inhibition of *miR-154-3p* (miR-154*) in mice led to a decrease in bone metastases along with an increase in survival [77,78]. Interestingly, Chen et al. proved that overexpression of *miR-154-5p* in nasopharyngeal carcinoma (NPC) cell line models suppresses their invasion and migration. Their mechanistic studies showed the regulatory function of the said miRNA which, by directly binding the 3′UTR of kinesin-like protein 14 (KIF14) mRNA, inhibits NPC metastasis [62].

Based on the target prediction tools, we selected 24 genes that were potential targets for *miR-154-5p*, and the expression levels of those genes were negatively correlated with the expression levels of *miR-154-5p* in the HNSCC patients. In that group VPS4B, LRRC1, DMXL1, NPEPPS, XPO7, LIN54, CEACAM7, CNOT4, C12ORF29, WAC, LIN9, RNF138, GNAI3, and DCAF16 had not been described previously in the context of the HNSCC nor EMT process. Our results indicated that those genes were co-expressed, had physical interactions, and were involved in the ESCRT, cell cycle, as well as viral replication.

The connections with HNSCC and EMT have been reported for ten genes. Zhang et al. observed that MYO6 (myosin VI) was upregulated in OSCC and was associated with the regulation of cell cycle progression and apoptosis [79]. In gastric cancer, MYO6 acts as a pro-metastatic or pro-EMT gene and is directly regulated by *miR-143*, as well as by *miR-145* [80]. Moreover, the identified potential target of *miR-154-5p* was OVOL1 (ovo-like transcriptional repressor 1) which was described as an important transcription factor in the mesenchymal-to-epithelial transition (MET) process [81,82]. It was proved that OVOL1 together with OVOL2 influenced the cellular phenotype by regulation of transcription factor ZEB1 and epithelial splicing regulatory protein 1 (ESRP1), which regulated mRNA splicing [82]. It has also been postulated that the family of transcription factors OVOL play a multiway function in cellular phenotype regulation by preventing EMT, driving MET, regulating the transition state (hybrid epithelial/mesenchymal), and maintaining a two-step process of transition EMT and MET [83]. We also observed that ABI1 (abl interactor 1) was negatively correlated with *miR-154-5p*. It has been indicated that low ABI1 expression was associated with metastasis and shorter survival of prostate cancer patients. Based on the in vitro studies, it has been revealed that ABI1 causes activation of the noncanonical WNT signaling and EMT pathways [84]. Another study showed that ABI1 has an inverse function, and its upregulation induced the EMT process and increased stem cell activity in breast cancer [85]. Furthermore, Fang et al. showed that the SOS1/EPS8/ABI1 complex was pivotal for ovarian cancer cells during the EMT process [86]. We also identified CDKN2B as a potential target. Reduced expression of CDKN2B was determined as a marker of disease recurrence in oral cancer [87]. In the case of hepatocellular carcinoma, reduced expression of CDKN1A and CDKN2B is connected with the EMT process, migration, and cell cycle progression [88]. Another identified target within this study was ESRP1. ESRP1 and ESRP2 play an important role in alternative splicing, and their expression is reduced during the EMT process [89]. It has been indicated that both were upregulated in dysplastic samples of OSCC, but their reduction was probably restricted in the cells that had a motile phenotype acquired during cancer invasion [90]. The next two *miR-154-5p* targets connected with EMT were CPEB3 and PLAGL2. Zhong et al. demonstrated that CPEB3 played a crucial role in the EMT process by regulation of the interaction between cancer cells and tumor-associated macrophages (TAMs). Its functions included inhibition of IL-6R/STAT3 signaling by binding to IL-6R mRNA in cancer cells as well as inhibition of the IL-6 molecule in TAMs by CPEB3. Finally, CPEB3 prevented secretion of CCL2 molecules in cancer cells and caused a reversion of the polarity of M2 macrophages [91]. Moreover, the role of CPEB3 in the progression of cervical cancer as well as in tumorigenesis and metastasis of lung adenocarcinoma was shown [92,93]. PLAGL2 (PLAG1-like zinc finger 2) seems to have an opposite role to CPEB3. It has been shown that PLAGL2 promoted EMT by USP37-mediated deubiquitination of Snail1 in gastric cancer [94], as well as β-catenin-dependent regulation of ZEB1 in colorectal cancer [95]. Additionally, higher levels of PLAGL2 were associated with metastasis [95] and finally with worse patient survival [94]. It should be noted that Wang et al. identified PLAGL2 as well as MAPKAPK5-AS1 as the direct targets of *miR-154-5p* and the MAPKAPK5-AS1/PLAGL2/HIF-1α signaling loop that is responsible for progression and metastasis of hepatocellular carcinoma [96]. We indicated that the next potential target for *miR-154-5p* was PTBP3. Wu et al. observed that PTBP3 was responsible for migration through the regulation of E-cadherin, influencing the EMT process, and its higher expression was associated with shorter survival in NSCLC [97]. However, Hou et al. demonstrated that PTBP3 was associated with lymph node metastasis, advanced stages, and poor OS of breast cancer patients. PTBP3 directly regulates the EMT process and cancer stem cells by binding to the 3′UTR of ZEB1 [98]. The last two potential *miR-154-5p* targets were TMPRSS11D and NSD2. Both of them have been described in HNSCC. The first one, TMPRSS11D, was one of the nine hub genes significantly correlated with HNSCC, and its higher levels were associated with better survival [99]. The other one, NSD2, was identified as altered in 6% of HNSCCs and is one of the lysine methyltransferases [100]. It has been observed that the low expression level of NSD2 was correlated with reduced OS of HPV-positive patients [101]. Nevertheless, NSD2 was overexpressed in patients with advanced cancer, who had poorly differentiated tumors, and its levels were higher than in normal epithelia. NSD2 directly regulated the transcription of NIMA-related kinase-7 (NEK7), which is a cell cycle regulator [102]. Moreover, lower levels of NSD2 caused a higher expression of the E-cadherin protein and decreased N-cadherin as well as vimentin. NSD2 interacts with TWIST1 and causes upregulation of H3K36me2, which, in turn, is manifested with EMT promotion [103].

Finally, our analysis of immunological features in groups of patients with low or high expression of both *miR-154* mature strands demonstrated some intriguing differences. The obtained results suggest that individuals with decreased *miR-154-5p* and *miR-154-3p* can be characterized by more favorable features. First of all, our assessment of the ESTIMATE score determined that these patients have lower tumor purity. Decreased values of this parameter were previously correlated with worse prognosis in, e.g., gastric cancers [104,105], colon cancer [106], and glioma [107]; however, it should be noted that the ESTIMATE score is calculated based on the stromal and immune scores. The large amount of stromal cells recruited from endogenous tissue promotes tumor growth and secrete many factors that influence angiogenesis, proliferation, and metastasis [108], though increased infiltration of immune cells is far more complicated to interpret.

Mandal et al. demonstrated that HNSCCs are among the most highly immune-infiltrated cancer types with an especially abundant T cell fraction, which indicates a better prognosis and response to immunotherapeutic approaches [109]. It should be emphasized that the positive impact is associated with high fractions of CD3+, CD8+, and regulatory T cells [109,110]. Interestingly, overexpression of CD3 is strongly correlated with better clinical outcomes of specifically HPV-negative individuals [110,111]. The increased counts of CD8+ lymphocytes do not bear an unequivocal prognostic function when correlated with the HPV infection status [110,112]. Nevertheless, Balermpas et al. showed that high levels of both CD3 and CD8 cells were significantly associated with improved distant metastasis-free survival (DMFS), which could imply the presence of a systemic immunosurveillance mechanism suppressing the development of micrometastases [110]. It is worth mentioning that da Silva et al. discovered a link between CD8+ T cells and expression of the genes coding CTA [113]. Protein products of these sequences can be found in only one healthy tissue—in the testis, and in a variety of tumor types, where they induce an immunological response [114]. It has been proven that in HNSCC, high levels of CTAs associated with a good prognosis are correlated with elevated counts of CD8+ T cells [113]. Our results also indicate the link between better OS and an increased level of CD8+ lymphocytes in low *miR-154* expression groups; however, the CTA scores are higher in the opposite groups of patients. We believe it is due to the fact that we worked on Thorssons’ CTA score values, which were assessed using a pool of various CT genes, without distinguishing them into groups related to good or poor prognosis [36].

Analysis of immune cell fractions infiltrating the tumor showed a decreased number of macrophages in the groups of patients with low expression of both *miR-154* strands. Kumar et al. proved that high density of TAMs, specifically of those with the M2-like phenotype, plays a crucial role in tumor progression, as well as metastasis, and correlates with worse clinicopathological features [115]. Interestingly, TAMs are characterized by downregulated expression of E-cadherin and elevated levels of mesenchymal markers, e.g., vimentin, Snail, and Slug, which could imply its involvement in the EMT [116]. A study by She et al. observed that CD163-positive macrophages (M2-like) play a pro-tumoral role and, when co-cultured with cancer cells, release TGF beta, epidermal growth factor (EGF) and upregulate ERK1/2, inducing tumor growth [116]. This type of macrophages inhibits proinflammatory M1-like ones through the production of, e.g., IL-10, TGF beta, as well as VEGF, leading to angiogenesis and tissue remodeling [117]. Additionally, IL-10 and TGF beta induce conversion of the M0 macrophages to the M2c type [118]. It should be emphasized that in HNSCC, pathways including TGF beta and FGFR are known to interact with EGFR signaling, which affects the wound healing process and could lead to EMT [119]. All of the above is reflected in our results, especially in the case of *miR-154-5p*. The high expression group had not only elevated macrophages, particularly M0 and M2, but also increased TGF beta response and elevated wound healing signatures, which confirms its substantial role in EMT and tumor progression. In addition, we determined that *miR-154-5p* reaches the highest expression level in the mesenchymal subtype of HNSCC in comparison to others, which seems to additionally corroborate the above statement. A recent study analyzing immune signatures in HNSCC proposed three different subtypes of these malignancies Immunity-H, -M, and -L (high, medium, and low, respectively). The first one was characterized by increased immune and stromal infiltration, low tumor purity, low stemness, as well as intratumor heterogeneity, genomic stability, and favorable prognosis in contrast to the immunity-L subtype [120]. These findings seem to support our obtained results. Yoshihara et al. suggested that in HNSCC, tumor purity is correlated with mutation rates, which is especially interesting while taking into account higher silent and non-silent mutation rates discovered by us in a group of individuals with decreased levels of *miR-154-5p* [47]. They also implied that in some low-purity neoplasms, reduction in the T > A substitutions indicates an impact of the tumor microenvironment on mutational processes or that mutation types can alter the stromal and immune infiltrations in tumors [47]. Furthermore, the above study suggested that the stromal expression pattern could overlap with the mesenchymal phenotype of tumors, which, in addition to the strong correlation with tumor purity, could result in incorrect classification of an increased presence of tumor-associated stroma as an EMT process [47]. Nonetheless, our knowledge of the *miR-154-5p* involvement in the abovementioned transition, along with the data obtained during this research, excludes the possibility of the aforementioned misinterpretation.

The Immunity-H subtype is associated with a higher rate of HPV infections in comparison to the Immunity-L subtype (40% vs. 22%) [120]. It has been proven that HPV-positive patients with oropharyngeal squamous cell carcinoma (OPSCC) who are current smokers or have tobacco use history tend to have a dysregulated ncRNA expression landscape, including downregulation of *miR-154-5p*, and unfavorable treatment outcomes [121]. A study by Huang et al. indicated that smokers and lung cancer patients also manifest with low levels of *miR-154* mature strands. Significant differences between these two groups of individuals and controls suggest the *miR-154-5p* diagnostic potential and imply an important role in the biology of cigarette smoke-induced lung cancer [122]. On the other hand, analysis of the *miR-154-3p* levels in plasma samples in groups of healthy smokers, individuals with lung granuloma and lung adenocarcinoma showed its overexpression in both types of patients. Those significant differences suggest that this molecule could help discriminate adenocarcinoma from lung granuloma in the future [123]. Interestingly, HNSCC patients with high molecular smoking signatures have an elevated number of mutations and decreased T cell infiltration, which is associated with local immunosuppression, low levels of cytotoxicity, and poor survival [109].

The broad network of *miR-154* interactions includes many molecular pathways essential for tumor development and progression. Further research to explore the role of this molecule, as well as its diagnostic and therapeutic potential, could yield results relevant to the quality and life expectancy of HNSCC patients.

In conclusion, our results indicate that *miR-154* plays an essential role in HNSCC development and progression, presumably through its influence on the Epithelial-to-Mesenchymal transition process and the regulation of phenotype of both cancer and immune cells. *miR-154-5p* targets the genes involved in the ESCRT, cell cycle, and viral replication, which are connected with the EMT process in other cancers as well. *miR-154* has a potential to be used as a biomarker describing clinical, molecular, and immunological features of HNSCC patients. Moreover, the implementation of *miR-154* in diagnosis and therapy might lead to the progress in the discovery of modern neoplasm treatment methods. However, it should be noted that the above data were obtained from in silico analyses and require further validation. *miR-154* is a molecule with a tremendous medical potential whose exploration may bring significant improvement in understanding HNSCC, and thus more effective treatment development.

## Figures and Tables

**Table 1:** The expression levels of *miR-154-5p* and *miR-154-3p* depended on the clinicopathological parameters in all the localizations of HNSCC; *t*-test or Mann–Whitney U test; *p* < 0.05 considered as significant and bolded.

Parameter	Group	*miR-154-5p*			*miR-154-3p*		
		Cases	Mean ± SEM	*p*	Cases	Mean ± SEM	*p*
Age	≤ 60	*n* = 250	1.919 ± 0.8643	0.2086	*n* = 247	1.491 ± 0.7910	0.2127
	> 61	*n* = 269	2.013 ± 0.8272		*n* = 264	1.582 ± 0.8511	
Gender	Female	*n* = 147	1.950 ± 0.7729	0.7350	*n* = 43	1.490 ± 0.7226	0.3860
	Male	*n* = 373	1.978 ± 0.8747		*n* = 369	1.560 ± 0.8606	
Alcohol	Yes	*n* = 337	1.959 ± 0.8285	0.9619	*n* = 331	1.539 ± 0.8437	0.8766
	No	*n* = 173	1.962 ± 0.8560		*n* = 171	1.527 ± 0.7874	
Smoking	No/ex	*n* = 203	1.925 ± 0.8307	0.2880	*n* = 201	1.529 ± 0.8028	0.8000
	Yes	*n* = 302	2.006 ± 0.8551		*n* = 296	1.549 ± 0.8442	
Cancer stage	I + II	*n* = 125	2.145 ± 0.8002	**0.0077**	*n = 123*	1.625 ± 0.7244	0.1674
	III + IV	*n* = 382	1.913 ± 0.8548		*n* = 376	1.510 ± 0.8291	
T-stage	T1 + T2	*n* = 189	2.001 ± 0.8369	0.5924	*n* = 189	1.511 ± 0.7406	0.5106
	T3 + T4	*n* = 316	1.959 ± 0.8497		*n* = 308	1.560 ± 0.8428	
N-stage	N0	*n* = 252	2.071 ± 0.8426	**0.0066**	*n* = 245	1.623 ± 0.8086	**0.0208**
	N1 + N2 + N3	*n* = 248	1.866 ± 0.8409		*n* = 246	1.455 ± 0.7938	
Grade	G1 + G2	*n* = 370	1.913 ± 0.8247	**0.0017**	*n* = 361	1.507 ± 0.7755	0.0589
	G3 + G4	*n* = 126	2.182 ± 0.8299		*n* = 127	1.667 ± 0.9413	
Perineural invasion	Positive	*n* = 171	2.090 ± 0.7819	0.0777	*n* = 169	1.682 ± 0.8359	0.1173
	Negative	*n* = 192	1.936 ± 0.8610		*n* = 188	1.538 ± 0.8889	
Lymph node neck dissection	Positive	*n* = 416	1.986 ± 0.8395	0.4054	*n* = 407	1.564 ± 0.8272	0.2810
	Negative	*n* = 102	1.908 ± 0.8814		*n* = 102	1.466 ± 0.8081	
HPV p16 status	Positive	*n* = 38	1.492 ± 0.9784	**0.0102**	*n* = 36	1.208 ± 0.7566	**0.0277**
	Negative	*n* = 65	1.943 ± 0.7552		*n* = 65	1.534 ± 0.6708	

## Data Availability

All the data are available online with common access. The data analyzed during this study are available from the corresponding author on reasonable request.

## Supplementary Materials

The following are available online at https://www.mdpi.com/article/10.3390/biomedicines9121894/s1, Table S1: List of processes based on the GSEA analysis of MSigDB gene sets enriched in HNSCC patients with lower and higher levels of *miR-154-5p* in all localization as well as depending on the localization in oral cavity, larynx or pharynx, Table S2: Correlation between *miR-154-5p* and 24 genes selected based on miRNA targets with score, function and interactions between targets using the GeneMANIA tool.
