# Supplementary material for: miR-154 Influences HNSCC Development and Progression through Regulation of the Epithelial-to-Mesenchymal Transition Process and Could Be Used as a Potential Biomarker
Source: Biomedicines. 2021 Dec 13;9(12):1894. doi: 10.3390/biomedicines9121894 (PMC8698850; doi:10.3390/biomedicines9121894)
Supplement: Supplementary file 1 [file biomedicines-09-01894-s001.zip › biomedicines-1423811-supplementary.pdf]

biomedicines

MDPI

Table S1. List of processes based on the GSEA analysis of MSigDB gene sets enriched in HNSCC patients with lower and higher levels of *miR-154-5p* in all localization as well as depending on the localization in oral cavity, larynx or pharynx. Only gene sets with nominal p < 0.05 were presented; SIZE - number of enriched genes in specified process, ES - enrichment score, NES - normalized enrichment score, FDR q-val - false discovery rate.

	NAME	SIZE	ES	NES	p-val	FDR q-val
**Localization**	**patients with low expression of miR-154-5p**					
All localizations	SINGH_KRAS_DEPENDENCY_SIGNATURE	20	-0.700524	-1.5727571	0.08045977	0.663567
	TBK1.DF_DN	258	-0.4619756	-1.56569	0.054054055	0.34981143
	**patients with high expression of miR-154-5p**					
	AKT_UP.V1_DN	179	0.5298579	2.0809271	0.0	0.010224129
	AKT_UP_MTOR_DN.V1_DN	177	0.42670274	1.8757594	0.0	0.093307704
	MTOR_UP.V1_DN	170	0.44283426	1.8146863	0.0	0.0974028
	ESC_J1_UP_EARLY.V1_DN	158	0.35960007	1.6201601	0.0019607844	0.18996853
	CRX_DN.V1_DN	127	0.44080538	1.7359952	0.003992016	0.13988577
	GCNP_SHH_UP_LATE.V1_DN	168	0.36878064	1.6192702	0.004008016	0.17715292
	ESC_V6.5_UP_EARLY.V1_DN	153	0.50741667	1.8727603	0.0040650405	0.06392599
	JNK_DN.V1_DN	174	0.4444347	1.7508625	0.00407332	0.14491369
	CYCLIN_D1_UP.V1_UP	178	0.38466176	1.6065778	0.0058708414	0.16989867
	GLI1_UP.V1_UP	21	0.51938635	1.6415216	0.009578544	0.23385362
	CAHOY_ASTROGLIAL	91	0.47678348	1.7227652	0.009652509	0.13629018
	CAHOY_OLIGODENDROCUTIC	85	0.38774285	1.5628906	0.01775148	0.16555104
	LEF1_UP.V1_UP	186	0.43609655	1.6494541	0.018832391	0.24566129
	KRAS.600.LUNG.BREAST_UP.V1_UP	267	0.3913184	1.6114745	0.019723866	0.17489475
	NRL_DN.V1_DN	121	0.35250053	1.5266105	0.021611001	0.17696068
	EIF4E_UP	88	0.4670218	1.6271266	0.021782178	0.1955343

BMI1_DN_MEL18_DN.V1_DN	137	0.4354125	1.6353568	0.024809161	0.2210591
CTIP_DN.V1_UP	120	0.42776752	1.5839401	0.026119404	0.15453193
PKCA_DN.V1_DN	146	0.34221587	1.4658694	0.029239766	0.20648196
KRAS.50_UP.V1_UP	47	0.49730524	1.6009626	0.031311154	0.16576183
CRX_NRL_DN.V1_DN	111	0.35360312	1.4673762	0.03206413	0.2162339
KRAS.AMP.LUNG_UP.V1_UP	130	0.40797198	1.5366397	0.03265306	0.17746533
WNT_UP.V1_UP	169	0.3281619	1.4002154	0.033203125	0.23158869
RB_P130_DN.V1_UP	116	0.41796416	1.5319397	0.033797216	0.17631269
CYCLIN_D1_KE_.V1_UP	182	0.32691562	1.4527216	0.034274194	0.20706365
KRAS.KIDNEY_UP.V1_UP	136	0.4982055	1.6296653	0.036821704	0.20976704
ATM_DN.V1_UP	140	0.36542484	1.4805324	0.03777336	0.21914394
ATF2_S_UP.V1_DN	176	0.42690405	1.5966355	0.038022812	0.15418139
P53_DN.V2_DN	141	0.3371459	1.415629	0.038383838	0.23218915
KRAS.600_UP.V1_UP	261	0.4146874	1.600918	0.03846154	0.15655284
CRX_NRL_DN.V1_UP	129	0.32568383	1.3895055	0.039447732	0.23613079
CAHOY_NEURONAL	94	0.433898	1.5554782	0.04016064	0.16692613
KRAS.300_UP.V1_UP	136	0.42623508	1.5853728	0.040229887	0.16072713
KRAS.PROSTATE_UP.V1_UP	126	0.3875258	1.4564825	0.046184737	0.21344519
BMI1_DN.V1_DN	128	0.37935737	1.4708267	0.048732944	0.21842751
ESC_V6.5_UP_LATE.V1_UP	170	0.3962382	1.5070236	0.04950495	0.19469327
HALLMARK_ANGIOGENESIS	36	0.7268911	1.9551228	0.0	0.028812956
HALLMARK_MYOGENESIS	198	0.70394534	2.0488024	0.0020449897	0.019075861
HALLMARK_EPITHELIAL_MESENCHYMAL_TRANSITION	194	0.7119223	1.9293958	0.003984064	0.027211443

	HALLMARK_COAGULATION	136	0.5151289	1.8114622	0.0075901328	0.06288996
	HALLMARK_PANCREAS_BETA_CELLS	40	0.47945312	1.6128231	0.02	0.22418033
Larynx	**patients with low expression of miR-154-5p**					
	HALLMARK_ESTROGEN_RESPONSE_EARLY	192	-0.3684512	-1.508271	0.032989692	1.0
	patients with high expression of miR-154-5p					
	AKT_UP_MTOR_DN.V1_DN	177	0.34209406	1.4716623	0.03269231	1.0
	AKT_UP.V1_DN	179	0.40278888	1.5882095	0.03992016	1.0
	HALLMARK_ANGIOGENESIS	36	0.59281003	1.6746945	0.03164557	0.36380783
	HALLMARK_MYOGENESIS	198	0.54568565	1.7289077	0.037109375	0.49452442
Pharynx	**patients with low expression of miR-154-5p**					
	DCA_UP.V1_DN	162	-0.32457164	-1.4496114	0.01934236	1.0
	ESC_J1_UP_EARLY.V1_UP	151	-0.32092696	-1.4028095	0.040935673	1.0
	HALLMARK_ALLOGRAFT_REJECTION	195	-0.63805974	-1.8395994	0.030927835	0.17024265
	**patients with high expression of miR-154-5p**					
	CRX_DN.V1_DN	127	0.42043114	1.6781394	0.010204081	0.3680043
	CRX_NRL_DN.V1_DN	111	0.3611626	1.5442594	0.010330578	0.5486713
	AKT_UP.V1_DN	179	0.43504387	1.6993006	0.016	0.6178347
	AKT_UP_MTOR_DN.V1_DN	177	0.3354621	1.4691406	0.031847134	0.5388127

	ESC_V6.5_UP_EARLY.V1_DN	153	0.45361865	1.6186346	0.03469388	0.42191383
	CAHOY_OLIGODENDROCUTIC	85	0.35082164	1.4489464	0.03526971	0.53511864
	ESC_J1_UP_LATE.V1_UP	176	0.37258855	1.4947804	0.035789475	0.5297214
	HALLMARK_MYOGENESIS	198	0.67982894	2.0338547	0.0041753654	0.006919477
	HALLMARK_EPITHELIAL_MESENCHYMAL_TRANSITION	194	0.6718212	1.7521929	0.02745098	0.11570975
**Oral cavity**	**patients with low expression of miR-154-5p**					
	RB_P130_DN.V1_DN	120	-0.48940206	-1.7510496	0.009345794	0.10240098
	TBK1.DF_DN	258	-0.5196189	-1.7025776	0.01622718	0.10524474
	SINGH_KRAS_DEPENDENCY_SIGNATURE	20	-0.76683635	-1.7550769	0.028397566	0.20084381
	**patients with high expression of miR-154-5p**					
	GCNP_SHH_UP_LATE.V1_DN	168	0.3742599	1.6583126	0.002020202	0.59006065
	RB_P130_DN.V1_UP	116	0.47475716	1.7137499	0.0020833334	1.0
	AKT_UP_MTOR_DN.V1_DN	177	0.36592835	1.6096988	0.0061728396	0.51515704
	CRX_DN.V1_UP	124	0.35605535	1.5382963	0.008350731	0.42748865
	SRC_UP.V1_UP	148	0.41562843	1.5529585	0.0139442235	0.5566733
	AKT_UP.V1_DN	179	0.43954867	1.7097505	0.018789144	0.5878324
	MTOR_UP.V1_DN	170	0.40486565	1.651626	0.0256917	0.46497825
	JNK_DN.V1_DN	174	0.4018756	1.5947857	0.02736842	0.48263174
	CRX_NRL_DN.V1_UP	129	0.3468031	1.4924229	0.035416666	0.5221901
	CYCLIN_D1_UP.V1_UP	178	0.34826428	1.4817967	0.035490606	0.47595248
	ESC_J1_UP_LATE.V1_DN	160	0.31664708	1.3999306	0.040983606	0.4827413

	ESC_V6.5_UP_LATE.V1_DN	162	0.31629592	1.4213246	0.04233871	0.52835584
	KRAS.AMP.LUNG_UP.V1_UP	130	0.40842712	1.5475217	0.047916666	0.5047504

Table S2. List of processes based on the GSEA analysis of MSigDB gene sets enriched in HNSCC patients with lower and higher levels of *miR-154-3p* in all localization as well as depending on the localization in oral cavity, larynx or pharynx. Only gene sets with nominal p < 0.05 were presented; SIZE - number of enriched genes in specified process, ES - enrichment score, NES - normalized enrichment score, FDR q-val - false discovery rate.

	NAME	SIZE	ES	NES	NOM p-val	FDR q-val
**Lokalization**	**patients with low expression of miR-154-3p**			No enrichment		
**All localizations**	**patients with high expression of miR-154-3p**					
	AKT_UP.V1_DN	186	0.56028676	2.1796565	0.0	0.0017931035
	ESC_V6.5_UP_LATE.V1_UP	187	0.4912813	1.9530286	0.0	0.024408296
	AKT_UP_MTOR_DN.V1_DN	183	0.44236442	1.9442787	0.0	0.01993367
	MTOR_UP.V1_DN	179	0.46488515	1.8907892	0.0	0.029356206
	ESC_V6.5_UP_EARLY.V1_DN	170	0.5393009	2.0287793	0.0018761726	0.015598844
	CRX_DN.V1_DN	133	0.43369287	1.7313417	0.0019569471	0.064509705
	CYCLIN_D1_UP.V1_UP	185	0.41847295	1.766866	0.0020120724	0.05659865
	CYCLIN_D1_KE_.V1_UP	187	0.36532766	1.6089052	0.0020120724	0.10600192
	GCNP_SHH_UP_LATE.V1_DN	175	0.38807258	1.6995656	0.0057142857	0.07046442
	LEF1_UP.V1_UP	193	0.48920476	1.8454154	0.005791506	0.039553046
	KRAS.600_UP.V1_UP	273	0.45381328	1.7537911	0.005859375	0.059982512
	CAHOY_ASTROGLIAL	100	0.50143784	1.8302763	0.0058708414	0.03419879
	NRL_DN.V1_DN	129	0.37102777	1.633271	0.0059405942	0.100295834
	BMI1_DN.V1_UP	144	0.56438947	1.8351312	0.007736944	0.038197946
	KRAS.300_UP.V1_UP	143	0.47411144	1.7534115	0.007874016	0.055062752
	KRAS.PROSTATE_UP.V1_UP	133	0.4167321	1.6173216	0.007874016	0.108538985
	PKCA_DN.V1_DN	160	0.36382353	1.5571125	0.008	0.120997645

	P53_DN.V2_DN	143	0.36883068	1.563393	0.008281574	0.11919013
	ATF2_S_UP.V1_DN	183	0.47763944	1.7724284	0.009578544	0.06050411
	CRX_NRL_DN.V1_DN	126	0.36530733	1.5539541	0.009823183	0.11645774
	ESC_J1_UP_LATE.V1_UP	190	0.43791965	1.7128996	0.0114722755	0.07128647
	JNK_DN.V1_DN	183	0.40268537	1.6128576	0.013618677	0.107554965
	SNF5_DN.V1_DN	161	0.397052	1.6032484	0.015384615	0.10261076
	KRAS.AMP.LUNG_UP.V1_UP	139	0.39752188	1.5398251	0.015968064	0.12231667
	ATM_DN.V1_UP	144	0.3898237	1.6068971	0.01622718	0.103437915
	CORDENONSI_YAP_CONSERVED_SIGNATURE	57	0.5127401	1.7085344	0.017274473	0.069210045
	CAHOY_OLIGODENDROCUTIC	97	0.382373	1.5325611	0.017964073	0.1257437
	ESC_J1_UP_EARLY.V1_DN	174	0.31309244	1.4448574	0.01908397	0.1757776
	WNT_UP.V1_UP	177	0.34359294	1.4747274	0.01984127	0.15992926
	PTEN_DN.V1_UP	185	0.41450268	1.5927682	0.022	0.10579497
	KRAS.KIDNEY_UP.V1_UP	142	0.49838185	1.647517	0.024242423	0.0982057
	CSR_LATE_UP.V1_DN	161	0.38936192	1.5541435	0.026768642	0.12012326
	BCAT.100_UP.V1_DN	38	0.44301105	1.5733078	0.026804123	0.11801403
	PTEN_DN.V2_DN	137	0.33657074	1.4035423	0.03422053	0.18592863
	MEL18_DN.V1_UP	140	0.5115615	1.6619292	0.036053132	0.093064725
	KRAS.50_UP.V1_UP	48	0.50891614	1.6336975	0.03646833	0.10493443
	RB_P130_DN.V1_UP	128	0.4099905	1.5316194	0.036734693	0.12316106
	PRC2_SUZ12_UP.V1_DN	182	0.34558478	1.4611349	0.03875969	0.16666754
	BCAT_BILD_ET_AL_UP	45	0.438524	1.5237733	0.03952569	0.12706403
	BCAT.100_UP.V1_UP	47	0.44722173	1.5408704	0.040152963	0.12528561
	BCAT_GDS748_DN	43	0.4005337	1.4635793	0.043010753	0.16798286
	HALLMARK_MYOGENESIS	198	0.5857865	1.8978672	0.010683761	0.12087148

	HALLMARK_EPITHELIAL_MESENCHYMAL_TRANSITION	194	0.6463002	1.8134077	0.027542373	0.14074229
**Larynx**	**patients with low expression of miR-154-3p**	No enrichment				
	**patients with high expression of miR-154-3p**					
	CSR_LATE_UP.V1_UP	170	0.5095815	1.8798832	0.004132231	0.08499931
	AKT_UP_MTOR_DN.V1_DN	183	0.3547783	1.5097848	0.015717093	0.4540008
	ESC_V6.5_UP_LATE.V1_UP	187	0.41751322	1.6434906	0.017509727	0.70383555
	BCAT_GDS748_UP	48	0.44833022	1.5904704	0.019305019	0.7317408
	SNF5_DN.V1_DN	161	0.36164638	1.5084386	0.024291499	0.41381618
	CRX_NRL_DN.V1_DN	126	0.3428042	1.4622862	0.04048583	0.41364855
	KRAS.600_UP.V1_UP	273	0.4309471	1.5710124	0.041257367	0.6375813
	KRAS.300_UP.V1_UP	143	0.43796596	1.5487969	0.046783626	0.60819215
	HALLMARK_MYOGENESIS	198	0.5857865	1.8978672	0.010683761	0.12087148
	HALLMARK_EPITHELIAL_MESENCHYMAL_TRANSITION	194	0.6463002	1.8134077	0.027542373	0.14074229
**Pharynx**	**patients with low expression of miR-154-3p**					
	HALLMARK_E2F_TARGETS	187	-0.6802637	-1.8862922	0.014112903	0.11118078
	HALLMARK_G2M_CHECKPOINT	184	-0.6064035	-1.8568065	0.018595042	0.07270152
	**patients with high expression of miR-154-3p**					
	ESC_J1_UP_LATE.V1_UP	190	0.48204732	1.929881	0.0	0.041719574
	BCAT.100_UP.V1_UP	47	0.5718491	1.8956203	0.0	0.035667643

	CRX_NRL_DN.V1_DN	126	0.42834833	1.86238	0.0	0.02767766
	CRX_DN.V1_DN	133	0.4646415	1.8566265	0.0	0.024174072
	NRL_DN.V1_DN	129	0.41368577	1.8391927	0.0	0.025369184
	AKT_UP_MTOR_DN.V1_DN	183	0.38696566	1.6563246	0.0	0.0771485
	AKT_UP.V1_DN	186	0.50033617	1.8796655	0.0018248175	0.023064781
	CAHOY_OLIGODENDROCUTIC	97	0.4253644	1.7329963	0.0018691589	0.048996754
	ESC_V6.5_UP_LATE.V1_UP	187	0.47258893	1.8950207	0.0019047619	0.023778427
	SNF5_DN.V1_DN	161	0.44967312	1.7536162	0.0019267823	0.04548479
	MTOR_UP.V1_DN	179	0.4247355	1.7566849	0.0036630037	0.048450958
	ESC_V6.5_UP_EARLY.V1_DN	170	0.5127685	1.8070983	0.0056179776	0.032437876
	LEF1_UP.V1_UP	193	0.46868858	1.7509977	0.0057803467	0.043366257
	YAP1_UP	46	0.46993658	1.6788923	0.0058365758	0.06923028
	WNT_UP.V1_UP	177	0.3788462	1.6375798	0.0058708414	0.07936641
	KRAS.KIDNEY_UP.V1_UP	142	0.5444543	1.7869693	0.0060362173	0.036202777
	CAHOY_ASTROGLIAL	100	0.47663546	1.7024447	0.015267176	0.059626777
	KRAS.300_UP.V1_UP	143	0.4651405	1.7122635	0.015748031	0.057191994
	CORDENONSI_YAP_CONSERVED_SIGNATURE	57	0.5108649	1.6654265	0.015936255	0.07502026
	KRAS.600_UP.V1_UP	273	0.42238936	1.6462033	0.018036073	0.08146983
	JNK_DN.V1_DN	183	0.3808899	1.5554901	0.018181818	0.14036986
	MEL18_DN.V1_DN	148	0.44720942	1.6443839	0.019723866	0.07875094
	GCNP_SHH_UP_LATE.V1_DN	175	0.32447812	1.4128036	0.02079395	0.22958317
	ATM_DN.V1_UP	144	0.36395836	1.5410647	0.022540983	0.14352724
	NOTCH_DN.V1_UP	185	0.34537467	1.4807234	0.023904383	0.1763045
	ATF2_S_UP.V1_DN	183	0.4366	1.6162044	0.0251938	0.0882855
	GLI1_UP.V1_UP	27	0.46683082	1.559212	0.027777778	0.14257443

	KRAS.50_UP.V1_UP	48	0.52359563	1.6350644	0.030947777	0.07757031
	BMI1_DN_MEL18_DN.V1_DN	146	0.4059437	1.542301	0.03187251	0.14784521
	KRAS.600.LUNG.BREAST_UP.V1_UP	274	0.36797923	1.4930128	0.038229376	0.18165754
	CTIP_DN.V1_UP	126	0.39599085	1.4975284	0.04477612	0.18251292
	PTEN_DN.V1_UP	185	0.3815244	1.4915613	0.046277665	0.1784513
	BCAT_BILD_ET_AL_UP	45	0.44675708	1.5034374	0.04681648	0.18095581
	CYCLIN_D1_KE_.V1_UP	187	0.33151716	1.4300984	0.04887218	0.22248046
	HALLMARK_MYOGENESIS	198	0.7145784	2.117106	0.0	0.0010399999
	HALLMARK_ANGIOGENESIS	36	0.69430226	1.8271418	0.001980198	0.03932752
	HALLMARK_EPITHELIAL_MESENCHYMAL_TRANSITION	194	0.72035646	1.8964407	0.0039138943	0.04117608
	HALLMARK_COAGULATION	136	0.52564484	1.8423983	0.006085193	0.04639429
	HALLMARK_APICAL_JUNCTION	194	0.4655951	1.6966884	0.013861386	0.074379586
	HALLMARK_UV_RESPONSE_DN	137	0.5291569	1.7341154	0.02434457	0.069298126
**Oral cavity**	**patients with low expression of miR-154-3p**					
	AKT_UP.V1_UP	166	-0.36235863	-1.5241708	0.020449897	0.53579515
	RB_P130_DN.V1_DN	136	-0.462385	-1.6441884	0.024	0.65249217
	AKT_UP_MTOR_DN.V1_UP	180	-0.32997704	-1.440209	0.032719836	0.552364
	SINGH_KRAS_DEPENDENCY_SIGNATURE_	20	-0.7355437	-1.6171035	0.037037037	0.40179473
	HALLMARK_ESTROGEN_RESPONSE_LATE	195	-0.3557498	-1.507586	0.021696253	1.0
	HALLMARK_HEME_METABOLISM	190	-0.30163595	-1.3503901	0.046692606	0.61580694

**patients with high expression of miR-154-3p**					
AKT_UP.V1_DN	186	0.50241196	1.9108553	0.0020618557	0.091749
AKT_UP_MTOR_DN.V1_DN	183	0.37051854	1.5987741	0.006355932	0.80869895
P53_DN.V2_DN	143	0.35756117	1.5130025	0.012765957	0.57937676
MTOR_UP.V1_DN	179	0.41138262	1.6778622	0.014344262	0.6059425
PTEN_DN.V2_DN	137	0.34919706	1.4653875	0.022680413	0.423244
CYCLIN_D1_UP.V1_UP	185	0.34569985	1.4742062	0.027027028	0.42829973
RB_P130_DN.V1_UP	128	0.43527794	1.553512	0.036437247	0.5809809
VEGF_A_UP.V1_UP	193	0.40866178	1.5551827	0.036659878	0.68731534
SNF5_DN.V1_UP	174	0.42084318	1.5565385	0.047325104	0.8492638
HALLMARK_MYOGENESIS	198	0.6216926	1.8754559	0.018789144	0.10435163
HALLMARK_ANGIOGENESIS	36	0.5797845	1.6619582	0.04008016	0.24042602
HALLMARK_EPITHELIAL_MESENCHYMAL_TRANSITION	194	0.5964984	1.7075882	0.047131147	0.2522565

Supplementary Table S2. Correlation between *miR-154-5p* and 24 genes selected based on miRNA targets with score, function and interactions between targets using the GeneMANIA tool.

**Correlation miR-154-5p and its target**					
**Analyzed pair**	**Spearman/Pearson R**	**R-coefficient**	**95% confidence interval**	**P value**	**Number of XY Pairs**
miR-154-5p vs. ABI1	Spearman r	-0.1954	-0.2853 to -0.1020	<0.0001	449
miR-154-5p vs. CPEB3	Spearman r	-0.1873	-0.2777 to -0.09376	<0.0001	449

miR-154-5p vs. DMXL1	Spearman r	-0.2121	-0.3012 to -0.1192	<0.0001	449
miR-154-5p vs. NPEPPS	Pearson r	-0.1895	-0.2772 to -0.09866	<0.0001	449
miR-154-5p vs. PLAGL2	Pearson r	-0.1601	-0.2489 to -0.06854	0.0007	449
miR-154-5p vs. CNOT4	Spearman r	-0.1821	-0.2727 to -0.08842	0.0001	449
miR-154-5p vs. LIN9	Spearman r	-0.1223	-0.2150 to -0.02733	0.0095	449
miR-154-5p vs. VPS4B	Spearman r	-0.3055	-0.3894 to -0.2165	<0.0001	449
miR-154-5p vs. WAC	Spearman r	-0.1242	-0.2169 to -0.02925	0.0084	449
miR-154-5p vs. CEACAM7	Spearman r	-0.2039	-0.2934 to -0.1108	<0.0001	449
miR-154-5p vs. LRRC1	Spearman r	-0.2211	-0.3099 to -0.1286	<0.0001	449
miR-154-5p vs. MYO6	Spearman r	-0.2353	-0.3233 to -0.1432	<0.0001	449
miR-154-5p vs. PTBP3	Spearman r	-0.1404	-0.2326 to -0.04575	0.0029	449
miR-154-5p vs. RNF138	Spearman r	-0.1013	-0.1947 to -0.006130	0.0318	449
miR-154-5p vs.	Spearman r	-0.1458	-0.2378 to -0.05126	0.002	449

C12ORF29					
miR-154-5p vs. CDKN2B	Spearman r	-0.1923	-0.2824 to -0.09886	<0.0001	449
miR-154-5p vs. DCAF16	Pearson r	-0.09533	-0.1862 to -0.002809	0.0435	449
miR-154-5p vs. ESRP1	Spearman r	-0.1884	-0.2786 to -0.09482	<0.0001	449
miR-154-5p vs. GNAI3	Spearman r	-0.1007	-0.1941 to -0.005451	0.033	449
miR-154-5p vs. LIN54	Pearson r	-0.1847	-0.2726 to -0.09381	<0.0001	449
miR-154-5p vs. NSD2	Pearson r	-0.09532	-0.1862 to -0.002806	0.0435	449
miR-154-5p vs. OVOL1	Spearman r	-0.1977	-0.2875 to -0.1044	<0.0001	449
miR-154-5p vs. TMPRSS11D	Spearman r	-0.1379	-0.2301 to -0.04320	0.0034	449
miR-154-5p vs. XPO7	Spearman r	-0.1862	-0.2766 to -0.09260	<0.0001	449
**Functions of miR-154-5p targets**					
Function			FDR	Genes in network	Genes in genome
ESCRT complex disassembly			0.02429992932934357	3	10
cell cycle checkpoint			0.0526712161109135	6	201
negative regulation of mitotic cell cycle			0.08558568597259571	6	263

positive regulation of viral life cycle	0.08558568597259571	3	25
regulation of viral release from host cell	0.08558568597259571	3	26
midbody	0.08558568597259571	3	21
virion assembly	0.08854277632818527	3	29
viral release from host cell	0.08854277632818527	3	29
multivesicular body organization	0.08854277632818527	3	30
negative regulation of cell cycle G1/S phase transition	0.08971922208701773	4	93
exit from host	0.08971922208701773	3	33
exit from host cell	0.08971922208701773	3	33

Score and function

Gene	Score	Functions			
LIN9	0.8147884106262913				
LRRC1	0.7570225744326464				
WAC	0.7514653433623196	cell cycle checkpoint			
DCAF16	0.7357887790082105				
C12orf29	0.7309620162356512				
CPEB3	0.7247154071581097				
OVOL1	0.7159273552468898				
CDKN2B	0.7057027545919609	cell cycle checkpoint, negative regulation of cell cycle G1/S phase transition, negative regulation of mitotic cell cycle			
CNOT4	0.6856385674944434	cell cycle checkpoint, negative regulation of cell cycle G1/S phase transition, negative regulation of mitotic cell cycle			
PLAGL2	0.6817377390752662				

NSD2	0.68137600097079				
PTBP3	0.6803369404968923				
NPEPPS	0.6738095573295986				
CEACAM7	0.6716646125926766				
XPO7	0.6671771642456521				
TMPRSS11D	0.6521309249840034				
DMXL1	0.6408571244012663				
ABI1	0.6360087013917106				
RNF138	0.633831587103936				
MYO6	0.6255285691983021				
ESRP1	0.6186632993783615				
LIN54	0.6093346904694159				
VPS4B	0.6017001241319717	ESCRT complex disassembly, exit from host, exit from host cell, midbody, multivesicular body organization, positive regulation of viral life cycle, regulation of viral release from host cell, viral release from host cell, virion assembly			
GNAI3	0.5644190534521365				
LIN52	0.04845389800461336				
LIN37	0.04000311206495183				
MYBL1	0.0378457639368982				
L3MBTL2	0.03733693259701737				
RBBP4	0.0335285503978775				
MYBL2	0.02959984250933967				
DYRK1A	0.013423408272858761				

DDB1	0.012356651079968806	exit from host, exit from host cell, positive regulation of viral life cycle, regulation of viral release from host cell, viral release from host cell			
MICAL3	0.011280595821396178	midbody			
MAU2	0.008645420886955413				
RECQL4	0.008607268645708599				
VTA1	0.008002275572721596	ESCRT complex disassembly, multivesicular body organization, virion assembly			
TOP2A	0.007159810872361727	cell cycle checkpoint, negative regulation of mitotic cell cycle			
RBL1	0.007020907488781258	negative regulation of cell cycle G1/S phase transition, negative regulation of mitotic cell cycle			
RELCH	0.006765726336106448				
RNF111	0.006410557774346515				
RALB	0.006392921164584897				
CDC25A	0.005997253606634945				
E2F1	0.005987795643748295	cell cycle checkpoint, negative regulation of cell cycle G1/S phase transition, negative regulation of mitotic cell cycle			
VPS4A	0.0057962397856759496	cell cycle checkpoint, ESCRT complex disassembly, exit from host, exit from host cell, midbody, multivesicular body organization, negative regulation of mitotic cell cycle, positive regulation of viral life cycle, regulation of viral release from host cell, viral release from host cell, virion assembly			
**Interactions between targets**					
**Gene 1**	**Gene 2**	**Weight**	**Network group**	**Network**	
NPEPPS	C12orf29	0.022279615	Co-expression	oth-Zlotnik-20	
TMPRSS11D	CDKN2B	0.015407225	Co-expression	oth-Zlotnik-20	
TMPRSS11D	CEACAM7	0.017072676	Co-expression	oth-Zlotnik-20	
ABI1	C12orf29	0.028698482	Co-expression	oth-Zlotnik-20	
ESRP1	LRRC1	0.008100562	Co-expression	oth-Zlotnik-20	
ESRP1	PTBP3	0.010739899	Co-expression	oth-Zlotnik-20	

VPS4B	LRRC1	0.01042017	Co-expression	oth-Zlotnik-20(	
VPS4B	ABI1	0.010710415	Co-expression	oth-Zlotnik-20(	
GNAI3	PTBP3	0.012533406	Co-expression	oth-Zlotnik-20(	
GNAI3	VPS4B	0.0058445884	Co-expression	oth-Zlotnik-20(	
MYBL2	MYBL1	0.012533161	Co-expression	oth-Zlotnik-20(	
VTA1	NPEPPS	0.023306785	Co-expression	oth-Zlotnik-20(	
RBL1	MYBL2	0.01107938	Co-expression	oth-Zlotnik-20(	
RELCH	C12orf29	0.022101806	Co-expression	oth-Zlotnik-20(	
RELCH	NPEPPS	0.018847946	Co-expression	oth-Zlotnik-20(	
RELCH	ABI1	0.022219187	Co-expression	oth-Zlotnik-20(	
RELCH	VPS4B	0.009669509	Co-expression	oth-Zlotnik-20(	
RNF111	WAC	0.008207716	Co-expression	oth-Zlotnik-20(	
RNF111	C12orf29	0.007489541	Co-expression	oth-Zlotnik-20(	
RNF111	NPEPPS	0.0069940635	Co-expression	oth-Zlotnik-20(	
RNF111	ABI1	0.007820658	Co-expression	oth-Zlotnik-20(	
RALB	NPEPPS	0.021908162	Co-expression	oth-Zlotnik-20(	
RALB	ABI1	0.029890688	Co-expression	oth-Zlotnik-20(	
RALB	VPS4B	0.0103175	Co-expression	oth-Zlotnik-20(	
RALB	RELCH	0.020367647	Co-expression	oth-Zlotnik-20(	
DCAF16	WAC	0.01693839	Co-expression	hen-Brown-20(	
PLAGL2	DCAF16	0.0098297475	Co-expression	hen-Brown-20(	
NSD2	DCAF16	0.013989579	Co-expression	hen-Brown-20(	
NSD2	PLAGL2	0.0083880285	Co-expression	hen-Brown-20(	
PTBP3	CDKN2B	0.009976122	Co-expression	hen-Brown-20(	
DYRK1A	RNF138	0.011046242	Co-expression	hen-Brown-20(	
RECQL4	NSD2	0.009081764	Co-expression	hen-Brown-20(	
RECQL4	MYBL2	0.006867876	Co-expression	hen-Brown-20(	
VTA1	ABI1	0.008112838	Co-expression	hen-Brown-20(	
TOP2A	NSD2	0.006955337	Co-expression	hen-Brown-20(	
TOP2A	MYBL2	0.0056763375	Co-expression	hen-Brown-20(	
TOP2A	RECQL4	0.0066834856	Co-expression	hen-Brown-20(	
RNF111	DMXL1	0.006378078	Co-expression	hen-Brown-20(	
RNF111	DYRK1A	0.014407489	Co-expression	hen-Brown-20(	

RALB	CEACAM7	0.0168126	Co-expression	hen-Brown-20(	
CDC25A	PLAGL2	0.0077637723	Co-expression	hen-Brown-20(	
CDC25A	NSD2	0.008300521	Co-expression	hen-Brown-20(	
CDC25A	TOP2A	0.0070079416	Co-expression	hen-Brown-20(	
E2F1	MYBL2	0.009568628	Co-expression	hen-Brown-20(	
E2F1	RECQL4	0.011047635	Co-expression	hen-Brown-20(	
E2F1	TOP2A	0.009636029	Co-expression	hen-Brown-20(	
CNOT4	CPEB3	0.010363249	Co-expression	'ang-Maris-20(	
NSD2	PLAGL2	0.006462226	Co-expression	'ang-Maris-20(	
ABI1	CPEB3	0.013267917	Co-expression	'ang-Maris-20(	
VPS4B	PTBP3	0.008485339	Co-expression	'ang-Maris-20(	
GNAI3	PLAGL2	0.011169017	Co-expression	'ang-Maris-20(	
MYBL1	NSD2	0.011091582	Co-expression	'ang-Maris-20(	
MYBL2	NSD2	0.0053544967	Co-expression	'ang-Maris-20(	
RECQL4	MYBL2	0.008053301	Co-expression	'ang-Maris-20(	
TOP2A	MYBL1	0.012757425	Co-expression	'ang-Maris-20(	
TOP2A	MYBL2	0.006396861	Co-expression	'ang-Maris-20(	
CDC25A	MYBL2	0.011774617	Co-expression	'ang-Maris-20(	
E2F1	TMPRSS11D	0.016067887	Co-expression	'ang-Maris-20(	
E2F1	MYBL2	0.009872481	Co-expression	'ang-Maris-20(	
XPO7	WAC	0.013202282	Co-expression	ıng-Cheung-2(	
DMXL1	CPEB3	0.0085755065	Co-expression	ıng-Cheung-2(	
ABI1	DMXL1	0.0042838175	Co-expression	ıng-Cheung-2(	
VPS4B	CPEB3	0.010885908	Co-expression	ıng-Cheung-2(	
VPS4B	ABI1	0.005750587	Co-expression	ıng-Cheung-2(	
GNAI3	C12orf29	0.00961275	Co-expression	ıng-Cheung-2(	
LIN37	C12orf29	0.007040282	Co-expression	ıng-Cheung-2(	
DYRK1A	VPS4B	0.011233574	Co-expression	ıng-Cheung-2(	
DDB1	OVOL1	0.009849815	Co-expression	ıng-Cheung-2(	
TOP2A	NSD2	0.013581877	Co-expression	ıng-Cheung-2(	
TOP2A	MYBL1	0.0072662365	Co-expression	ıng-Cheung-2(	
TOP2A	RECQL4	0.0078024035	Co-expression	ıng-Cheung-2(	
RNF111	GNAI3	0.030458715	Co-expression	ıng-Cheung-2(	

CPEB3	C12orf29	0.0096941395	Co-expression	d-Nevins-2006	
DMXL1	CPEB3	0.011898731	Co-expression	d-Nevins-2006	
MYO6	DMXL1	0.024418302	Co-expression	d-Nevins-2006	
RBBP4	NSD2	0.021445418	Co-expression	d-Nevins-2006	
DYRK1A	ABI1	0.015365416	Co-expression	d-Nevins-2006	
TOP2A	MYBL2	0.008425637	Co-expression	d-Nevins-2006	
PLAGL2	CDKN2B	0.02327909	Co-expression	swamy-Golub	
ABI1	PTBP3	0.009284825	Co-expression	swamy-Golub	
RNF138	ABI1	0.003808728	Co-expression	swamy-Golub	
DDB1	RBBP4	0.005874628	Co-expression	swamy-Golub	
VTA1	ABI1	0.006134585	Co-expression	swamy-Golub	
RNF111	RELCH	0.013163213	Co-expression	swamy-Golub	
RNF138	C12orf29	0.01146754	Co-expression	bin-Giordano-	
ESRP1	OVOL1	0.011730528	Co-expression	bin-Giordano-	
ESRP1	PTBP3	0.012982073	Co-expression	bin-Giordano-	
VPS4B	DMXL1	0.01086625	Co-expression	bin-Giordano-	
MYBL1	NSD2	0.0068347766	Co-expression	bin-Giordano-	
RBBP4	XPO7	0.012448914	Co-expression	bin-Giordano-	
RECQL4	LIN37	0.016968794	Co-expression	bin-Giordano-	
TOP2A	NSD2	0.004781825	Co-expression	bin-Giordano-	
TOP2A	MYBL1	0.004119247	Co-expression	bin-Giordano-	
RBL1	RECQL4	0.012895215	Co-expression	bin-Giordano-	
RNF111	VPS4B	0.013588887	Co-expression	bin-Giordano-	
RALB	PTBP3	0.02232499	Co-expression	bin-Giordano-	
CDC25A	MYBL2	0.012118931	Co-expression	bin-Giordano-	
E2F1	XPO7	0.009310313	Co-expression	bin-Giordano-	
E2F1	RECQL4	0.010547243	Co-expression	bin-Giordano-	
E2F1	CDC25A	0.012645964	Co-expression	bin-Giordano-	
RNF138	DMXL1	0.0074333134	Co-expression	js-Rutgeerts-2(	
ESRP1	NSD2	0.006375138	Co-expression	js-Rutgeerts-2(	
ESRP1	CEACAM7	0.0025032891	Co-expression	js-Rutgeerts-2(	
LIN54	RNF138	0.007996068	Co-expression	js-Rutgeerts-2(	
VPS4B	LRRC1	0.0145602925	Co-expression	js-Rutgeerts-2(	

RELCH	ESRP1	0.006304463	Co-expression	js-Rutgeerts-2(	
RALB	NPEPPS	0.009858193	Co-expression	js-Rutgeerts-2(	
RALB	GNAI3	0.013574837	Co-expression	js-Rutgeerts-2(	
ABI1	WAC	0.010881725	Co-expression	ıng-de Kok-20	
RNF138	PTBP3	0.016972717	Co-expression	ıng-de Kok-20	
MYBL2	LIN9	0.010876305	Co-expression	ıng-de Kok-20	
MYBL2	MYBL1	0.013747675	Co-expression	ıng-de Kok-20	
DDB1	MYBL2	0.009490002	Co-expression	ıng-de Kok-20	
RECQL4	MYBL1	0.008707146	Co-expression	ıng-de Kok-20	
RECQL4	MAU2	0.0048077498	Co-expression	ıng-de Kok-20	
RBL1	LIN52	0.022730982	Co-expression	ıng-de Kok-20	
RNF111	CNOT4	0.022585403	Co-expression	ıng-de Kok-20	
RNF111	VPS4B	0.022444284	Co-expression	ıng-de Kok-20	
CDC25A	LIN52	0.015007587	Co-expression	ıng-de Kok-20	
E2F1	RBBP4	0.009021838	Co-expression	ıng-de Kok-20	
E2F1	RECQL4	0.002568588	Co-expression	ıng-de Kok-20	
E2F1	CDC25A	0.005476635	Co-expression	ıng-de Kok-20	
XPO7	CNOT4	0.007284655	Co-expression	ɔcenti-Brown-2	
GNAI3	ABI1	0.008847705	Co-expression	ɔcenti-Brown-2	
GNAI3	RNF138	0.005960107	Co-expression	ɔcenti-Brown-2	
GNAI3	VPS4B	0.00716698	Co-expression	ɔcenti-Brown-2	
MAU2	DCAF16	0.016808547	Co-expression	ɔcenti-Brown-2	
TOP2A	NSD2	0.007172603	Co-expression	ɔcenti-Brown-2	
TOP2A	RECQL4	0.009080315	Co-expression	ɔcenti-Brown-2	
RNF111	DCAF16	0.013473146	Co-expression	ɔcenti-Brown-2	
RNF111	GNAI3	0.0037349486	Co-expression	ɔcenti-Brown-2	
CDC25A	RECQL4	0.02032586	Co-expression	ɔcenti-Brown-2	
E2F1	NSD2	0.013699018	Co-expression	ɔcenti-Brown-2	
E2F1	RECQL4	0.01773359	Co-expression	ɔcenti-Brown-2	
E2F1	TOP2A	0.007254075	Co-expression	ɔcenti-Brown-2	
E2F1	RBL1	0.013772248	Co-expression	ɔcenti-Brown-2	
E2F1	CDC25A	0.021636382	Co-expression	ɔcenti-Brown-2	
MYO6	NSD2	0.009504871	Co-expression	on-Shaughnes	

VPS4B	PTBP3	0.01384683	Co-expression	on-Shaughnes	
MYBL1	NSD2	0.00996977	Co-expression	on-Shaughnes	
TOP2A	MYBL2	0.0041000918	Co-expression	on-Shaughnes	
CDC25A	MYBL2	0.0110226935	Co-expression	on-Shaughnes	
CDC25A	TOP2A	0.008536423	Co-expression	on-Shaughnes	
PLAGL2	CPEB3	0.015035562	Co-expression	ieger-Chu-200	
XPO7	NSD2	0.0044691483	Co-expression	ieger-Chu-200	
RBBP4	NSD2	0.0077157044	Co-expression	ieger-Chu-200	
MYBL2	NSD2	0.0041634957	Co-expression	ieger-Chu-200	
DYRK1A	ABI1	0.014046682	Co-expression	ieger-Chu-200	
DDB1	NSD2	0.0058653946	Co-expression	ieger-Chu-200	
TOP2A	XPO7	0.00877425	Co-expression	ieger-Chu-200	
RBL1	NSD2	0.00518808	Co-expression	ieger-Chu-200	
CDC25A	MYBL2	0.013165738	Co-expression	ieger-Chu-200	
MYO6	LRRC1	0.0046979245	Co-expression	llon-McKay-2(	
VPS4B	ABI1	0.017125152	Co-expression	llon-McKay-2(	
RBBP4	GNAI3	0.0097209215	Co-expression	llon-McKay-2(	
DDB1	NPEPPS	0.017561503	Co-expression	llon-McKay-2(	
MAU2	DCAF16	0.009380774	Co-expression	llon-McKay-2(	
MAU2	MICAL3	0.010434723	Co-expression	llon-McKay-2(	
VTA1	GNAI3	0.009623963	Co-expression	llon-McKay-2(	
RBL1	RECQL4	0.011747229	Co-expression	llon-McKay-2(	
RELCH	GNAI3	0.008382405	Co-expression	llon-McKay-2(	
RNF111	ABI1	0.005608169	Co-expression	llon-McKay-2(	
E2F1	RECQL4	0.0058196057	Co-expression	llon-McKay-2(	
E2F1	RBL1	0.013387715	Co-expression	llon-McKay-2(	
CEACAM7	LRRC1	0.019929463	Co-localization	on-Shoemaker	
ESRP1	LRRC1	0.011078517	Co-localization	on-Shoemaker	
ESRP1	CEACAM7	0.00807846	Co-localization	on-Shoemaker	
VPS4B	CEACAM7	0.014546804	Co-localization	on-Shoemaker	
VPS4B	ESRP1	0.009720429	Co-localization	on-Shoemaker	
GNAI3	PTBP3	0.013962214	Co-localization	on-Shoemaker	
RBBP4	NSD2	0.0076454827	Co-localization	on-Shoemaker	

RBL1	NSD2	0.010254401	Co-localization	on-Shoemaker	
RBL1	MYBL2	0.010436047	Co-localization	on-Shoemaker	
RNF111	WAC	0.024710106	Co-localization	on-Shoemaker	
RNF111	DMXL1	0.02159738	Co-localization	on-Shoemaker	
RNF111	L3MBTL2	0.028319264	Co-localization	on-Shoemaker	
RALB	MYO6	0.018514829	Co-localization	on-Shoemaker	
E2F1	RBBP4	0.0075191325	Co-localization	on-Shoemaker	
E2F1	RBL1	0.010516205	Co-localization	on-Shoemaker	
WAC	LRRC1	0.0012887164	Genetic Interactions	Lin-Smith-2010	
NSD2	CNOT4	0.00088065525	Genetic Interactions	Lin-Smith-2010	
PTBP3	WAC	0.00097053056	Genetic Interactions	Lin-Smith-2010	
XPO7	LRRC1	0.0012810407	Genetic Interactions	Lin-Smith-2010	
DMXL1	LIN9	0.0014897025	Genetic Interactions	Lin-Smith-2010	
DMXL1	C12orf29	0.0022103342	Genetic Interactions	Lin-Smith-2010	
DMXL1	OVOL1	0.0036268476	Genetic Interactions	Lin-Smith-2010	
DMXL1	CNOT4	0.0011811948	Genetic Interactions	Lin-Smith-2010	
RNF138	LIN9	0.0019133738	Genetic Interactions	Lin-Smith-2010	
VPS4B	CPEB3	0.001302679	Genetic Interactions	Lin-Smith-2010	
LIN37	XPO7	0.0020766188	Genetic Interactions	Lin-Smith-2010	
DYRK1A	C12orf29	0.0010314529	Genetic Interactions	Lin-Smith-2010	
DYRK1A	CPEB3	0.0006483199	Genetic Interactions	Lin-Smith-2010	
DYRK1A	PTBP3	0.00043667635	Genetic Interactions	Lin-Smith-2010	
DYRK1A	RNF138	0.00088413444	Genetic Interactions	Lin-Smith-2010	
DYRK1A	RBBP4	0.00044498104	Genetic Interactions	Lin-Smith-2010	
DDB1	WAC	0.002527239	Genetic Interactions	Lin-Smith-2010	
MICAL3	CDKN2B	0.0031327952	Genetic Interactions	Lin-Smith-2010	
MICAL3	DMXL1	0.0021905967	Genetic Interactions	Lin-Smith-2010	
MAU2	DMXL1	0.004889198	Genetic Interactions	Lin-Smith-2010	
TOP2A	MYO6	0.0021287168	Genetic Interactions	Lin-Smith-2010	
RBL1	LIN9	0.0017601575	Genetic Interactions	Lin-Smith-2010	
RBL1	RNF138	0.0022386124	Genetic Interactions	Lin-Smith-2010	
RELCH	PTBP3	0.00095425546	Genetic Interactions	Lin-Smith-2010	
RNF111	DYRK1A	0.00068071636	Genetic Interactions	Lin-Smith-2010	

RALB	NPEPPS	0.0021395036	Genetic Interactions	Lin-Smith-201[illegible]	
RALB	TMPRSS11D	0.0019826521	Genetic Interactions	Lin-Smith-201[illegible]	
RALB	DYRK1A	0.00050430605	Genetic Interactions	Lin-Smith-201[illegible]	
E2F1	XPO7	0.0023431063	Genetic Interactions	Lin-Smith-201[illegible]	
VPS4A	CNOT4	0.0019036788	Genetic Interactions	Lin-Smith-201[illegible]	
LIN54	LIN9	0.089336544	Pathway	REACTOME	
LIN52	LIN9	0.089336544	Pathway	REACTOME	
LIN52	LIN54	0.089336544	Pathway	REACTOME	
LIN37	LIN9	0.089336544	Pathway	REACTOME	
LIN37	LIN54	0.089336544	Pathway	REACTOME	
LIN37	LIN52	0.089336544	Pathway	REACTOME	
RBBP4	LIN9	0.05614493	Pathway	REACTOME	
RBBP4	LIN54	0.05614493	Pathway	REACTOME	
RBBP4	LIN52	0.05614493	Pathway	REACTOME	
RBBP4	LIN37	0.05614493	Pathway	REACTOME	
DYRK1A	LIN9	0.17221223	Pathway	REACTOME	
DYRK1A	LIN54	0.17221223	Pathway	REACTOME	
DYRK1A	LIN52	0.17221223	Pathway	REACTOME	
DYRK1A	LIN37	0.17221223	Pathway	REACTOME	
DYRK1A	RBBP4	0.10822944	Pathway	REACTOME	
VTA1	VPS4B	0.18933201	Pathway	REACTOME	
TOP2A	LIN9	0.09858862	Pathway	REACTOME	
TOP2A	LIN54	0.09858862	Pathway	REACTOME	
TOP2A	LIN52	0.09858862	Pathway	REACTOME	
TOP2A	LIN37	0.09858862	Pathway	REACTOME	
TOP2A	RBBP4	0.061959546	Pathway	REACTOME	
RBL1	LIN9	0.09421307	Pathway	REACTOME	
RBL1	LIN54	0.09421307	Pathway	REACTOME	
RBL1	LIN52	0.09421307	Pathway	REACTOME	
RBL1	LIN37	0.09421307	Pathway	REACTOME	
RBL1	TOP2A	0.103970185	Pathway	REACTOME	
CDC25A	LIN9	0.08592139	Pathway	REACTOME	
CDC25A	LIN54	0.08592139	Pathway	REACTOME	

CDC25A	LIN52	0.08592139	Pathway	REACTOME	
CDC25A	LIN37	0.08592139	Pathway	REACTOME	
CDC25A	RBBP4	0.053998627	Pathway	REACTOME	
CDC25A	TOP2A	0.094819784	Pathway	REACTOME	
CDC25A	RBL1	0.0906115	Pathway	REACTOME	
E2F1	LIN9	0.0822111	Pathway	REACTOME	
E2F1	LIN54	0.0822111	Pathway	REACTOME	
E2F1	LIN52	0.0822111	Pathway	REACTOME	
E2F1	LIN37	0.0822111	Pathway	REACTOME	
E2F1	TOP2A	0.09072524	Pathway	REACTOME	
E2F1	CDC25A	0.07906834	Pathway	REACTOME	
VPS4A	VPS4B	0.18933201	Pathway	REACTOME	
VPS4A	VTA1	0.21876583	Pathway	REACTOME	
LIN54	LIN9	0.37796447	Physical Interactions	:chins-Peters-2	
LIN52	LIN54	0.37796447	Physical Interactions	:chins-Peters-2	
LIN37	LIN54	0.37796447	Physical Interactions	:chins-Peters-2	
MYBL1	LIN54	0.37796447	Physical Interactions	:chins-Peters-2	
L3MBTL2	LIN54	0.37796447	Physical Interactions	:chins-Peters-2	
RBBP4	LIN54	0.37796447	Physical Interactions	:chins-Peters-2	
MYBL2	LIN54	0.37796447	Physical Interactions	:chins-Peters-2	
DDB1	DCAF16	0.19569455	Physical Interactions	:chins-Peters-2	
MICAL3	GNAI3	0.15001437	Physical Interactions	:chins-Peters-2	
MAU2	GNAI3	0.11073357	Physical Interactions	:chins-Peters-2	
RECQL4	GNAI3	0.12551126	Physical Interactions	:chins-Peters-2	

LIN54	LIN9	0.060034964	Physical Interactions	IREF-reactome	
LIN52	LIN9	0.060034964	Physical Interactions	IREF-reactome	
LIN52	LIN54	0.060034964	Physical Interactions	IREF-reactome	
LIN37	LIN9	0.060034964	Physical Interactions	IREF-reactome	
LIN37	LIN54	0.060034964	Physical Interactions	IREF-reactome	
LIN37	LIN52	0.060034964	Physical Interactions	IREF-reactome	
RBBP4	LIN9	0.026713608	Physical Interactions	IREF-reactome	
RBBP4	LIN54	0.026713608	Physical Interactions	IREF-reactome	
RBBP4	LIN52	0.026713608	Physical Interactions	IREF-reactome	
RBBP4	LIN37	0.026713608	Physical Interactions	IREF-reactome	
MYBL2	LIN9	0.075245954	Physical Interactions	IREF-reactome	
MYBL2	LIN54	0.075245954	Physical Interactions	IREF-reactome	
MYBL2	LIN52	0.075245954	Physical Interactions	IREF-reactome	
MYBL2	LIN37	0.075245954	Physical Interactions	IREF-reactome	
MYBL2	RBBP4	0.033482004	Physical Interactions	IREF-reactome	
DYRK1A	LIN9	0.10136444	Physical Interactions	IREF-reactome	
DYRK1A	LIN54	0.10136444	Physical Interactions	IREF-reactome	
DYRK1A	LIN52	0.10136444	Physical Interactions	IREF-reactome	

DYRK1A	LIN37	0.10136444	Physical Interactions	IREF-reactome	
DYRK1A	RBBP4	0.045103885	Physical Interactions	IREF-reactome	
VTA1	VPS4B	0.042756777	Physical Interactions	IREF-reactome	
TOP2A	LIN9	0.10136444	Physical Interactions	IREF-reactome	
TOP2A	LIN54	0.10136444	Physical Interactions	IREF-reactome	
TOP2A	LIN52	0.10136444	Physical Interactions	IREF-reactome	
TOP2A	LIN37	0.10136444	Physical Interactions	IREF-reactome	
TOP2A	RBBP4	0.045103885	Physical Interactions	IREF-reactome	
RBL1	LIN9	0.058857713	Physical Interactions	IREF-reactome	
RBL1	LIN54	0.058857713	Physical Interactions	IREF-reactome	
RBL1	LIN52	0.058857713	Physical Interactions	IREF-reactome	
RBL1	LIN37	0.058857713	Physical Interactions	IREF-reactome	
RBL1	RBBP4	0.026189769	Physical Interactions	IREF-reactome	
RBL1	MYBL2	0.073770426	Physical Interactions	IREF-reactome	
CDC25A	LIN9	0.018863164	Physical Interactions	IREF-reactome	
CDC25A	LIN54	0.018863164	Physical Interactions	IREF-reactome	
CDC25A	LIN52	0.018863164	Physical Interactions	IREF-reactome	
CDC25A	LIN37	0.018863164	Physical Interactions	IREF-reactome	

CDC25A	RBBP4	0.008393494	Physical Interactions	IREF-reactome	
E2F1	LIN9	0.03446678	Physical Interactions	IREF-reactome	
E2F1	LIN54	0.03446678	Physical Interactions	IREF-reactome	
E2F1	LIN52	0.03446678	Physical Interactions	IREF-reactome	
E2F1	LIN37	0.03446678	Physical Interactions	IREF-reactome	
E2F1	RBBP4	0.0153365955	Physical Interactions	IREF-reactome	
E2F1	RBL1	0.033790905	Physical Interactions	IREF-reactome	
VPS4A	VPS4B	0.042756777	Physical Interactions	IREF-reactome	
VPS4A	VTA1	0.042756777	Physical Interactions	IREF-reactome	
LIN54	LIN9	0.060034964	Physical Interactions	astrik-Stein-20	
LIN52	LIN9	0.060034964	Physical Interactions	astrik-Stein-20	
LIN52	LIN54	0.060034964	Physical Interactions	astrik-Stein-20	
LIN37	LIN9	0.060034964	Physical Interactions	astrik-Stein-20	
LIN37	LIN54	0.060034964	Physical Interactions	astrik-Stein-20	
LIN37	LIN52	0.060034964	Physical Interactions	astrik-Stein-20	
RBBP4	LIN9	0.026713608	Physical Interactions	astrik-Stein-20	
RBBP4	LIN54	0.026713608	Physical Interactions	astrik-Stein-20	
RBBP4	LIN52	0.026713608	Physical Interactions	astrik-Stein-20	

RBBP4	LIN37	0.026713608	Physical Interactions	astrik-Stein-20	
MYBL2	LIN9	0.075245954	Physical Interactions	astrik-Stein-20	
MYBL2	LIN54	0.075245954	Physical Interactions	astrik-Stein-20	
MYBL2	LIN52	0.075245954	Physical Interactions	astrik-Stein-20	
MYBL2	LIN37	0.075245954	Physical Interactions	astrik-Stein-20	
MYBL2	RBBP4	0.033482004	Physical Interactions	astrik-Stein-20	
DYRK1A	LIN9	0.10136444	Physical Interactions	astrik-Stein-20	
DYRK1A	LIN54	0.10136444	Physical Interactions	astrik-Stein-20	
DYRK1A	LIN52	0.10136444	Physical Interactions	astrik-Stein-20	
DYRK1A	LIN37	0.10136444	Physical Interactions	astrik-Stein-20	
DYRK1A	RBBP4	0.045103885	Physical Interactions	astrik-Stein-20	
VTA1	VPS4B	0.042756777	Physical Interactions	astrik-Stein-20	
TOP2A	LIN9	0.10136444	Physical Interactions	astrik-Stein-20	
TOP2A	LIN54	0.10136444	Physical Interactions	astrik-Stein-20	
TOP2A	LIN52	0.10136444	Physical Interactions	astrik-Stein-20	
TOP2A	LIN37	0.10136444	Physical Interactions	astrik-Stein-20	
TOP2A	RBBP4	0.045103885	Physical Interactions	astrik-Stein-20	
RBL1	LIN9	0.058857713	Physical Interactions	astrik-Stein-20	

RBL1	LIN54	0.058857713	Physical Interactions	astrik-Stein-20	
RBL1	LIN52	0.058857713	Physical Interactions	astrik-Stein-20	
RBL1	LIN37	0.058857713	Physical Interactions	astrik-Stein-20	
RBL1	RBBP4	0.026189769	Physical Interactions	astrik-Stein-20	
RBL1	MYBL2	0.073770426	Physical Interactions	astrik-Stein-20	
CDC25A	LIN9	0.018863164	Physical Interactions	astrik-Stein-20	
CDC25A	LIN54	0.018863164	Physical Interactions	astrik-Stein-20	
CDC25A	LIN52	0.018863164	Physical Interactions	astrik-Stein-20	
CDC25A	LIN37	0.018863164	Physical Interactions	astrik-Stein-20	
CDC25A	RBBP4	0.008393494	Physical Interactions	astrik-Stein-20	
E2F1	LIN9	0.03446678	Physical Interactions	astrik-Stein-20	
E2F1	LIN54	0.03446678	Physical Interactions	astrik-Stein-20	
E2F1	LIN52	0.03446678	Physical Interactions	astrik-Stein-20	
E2F1	LIN37	0.03446678	Physical Interactions	astrik-Stein-20	
E2F1	RBBP4	0.0153365955	Physical Interactions	astrik-Stein-20	
E2F1	RBL1	0.033790905	Physical Interactions	astrik-Stein-20	
VPS4A	VPS4B	0.042756777	Physical Interactions	astrik-Stein-20	
VPS4A	VTA1	0.042756777	Physical Interactions	astrik-Stein-20	

LIN54	LIN9	0.10755723	Physical Interactions	SMALL-SCALE	
LIN52	LIN9	0.15971665	Physical Interactions	SMALL-SCALE	
LIN52	LIN54	0.15396184	Physical Interactions	SMALL-SCALE	
LIN37	LIN9	0.10612605	Physical Interactions	SMALL-SCALE	
LIN37	LIN54	0.10230218	Physical Interactions	SMALL-SCALE	
LIN37	LIN52	0.15191317	Physical Interactions	SMALL-SCALE	
MYBL1	LIN9	0.17815623	Physical Interactions	SMALL-SCALE	
MYBL1	LIN54	0.171737	Physical Interactions	SMALL-SCALE	
MYBL1	LIN37	0.16945182	Physical Interactions	SMALL-SCALE	
RBBP4	LIN9	0.021426795	Physical Interactions	SMALL-SCALE	
RBBP4	RNF138	0.02764963	Physical Interactions	SMALL-SCALE	
RBBP4	LIN54	0.020654757	Physical Interactions	SMALL-SCALE	
RBBP4	LIN52	0.030671189	Physical Interactions	SMALL-SCALE	
RBBP4	LIN37	0.020379918	Physical Interactions	SMALL-SCALE	
MYBL2	LIN9	0.064028874	Physical Interactions	SMALL-SCALE	
MYBL2	LIN54	0.06172182	Physical Interactions	SMALL-SCALE	
MYBL2	LIN52	0.09165355	Physical Interactions	SMALL-SCALE	
MYBL2	LIN37	0.06090053	Physical Interactions	SMALL-SCALE	

MYBL2	RBBP4	0.012295785	Physical Interactions	MALL-SCAL	
DYRK1A	LIN52	0.06960841	Physical Interactions	MALL-SCAL	
DDB1	DCAF16	0.041741073	Physical Interactions	MALL-SCAL	
DDB1	MYO6	0.0045392253	Physical Interactions	MALL-SCAL	
DDB1	RBBP4	0.0025839908	Physical Interactions	MALL-SCAL	
RECQL4	DDB1	0.007584225	Physical Interactions	MALL-SCAL	
TOP2A	NSD2	0.00819744	Physical Interactions	MALL-SCAL	
RBL1	LIN9	0.039356906	Physical Interactions	MALL-SCAL	
RBL1	LIN54	0.037938822	Physical Interactions	MALL-SCAL	
RBL1	LIN52	0.056337085	Physical Interactions	MALL-SCAL	
RBL1	LIN37	0.037433993	Physical Interactions	MALL-SCAL	
RBL1	MYBL2	0.022584995	Physical Interactions	MALL-SCAL	
RBL1	DYRK1A	0.0171527	Physical Interactions	MALL-SCAL	
RNF111	ESRP1	0.2635499	Physical Interactions	MALL-SCAL	
E2F1	RBBP4	0.004274237	Physical Interactions	MALL-SCAL	
E2F1	MYBL2	0.012772539	Physical Interactions	MALL-SCAL	
E2F1	DDB1	0.0026841816	Physical Interactions	MALL-SCAL	
E2F1	RBL1	0.007850952	Physical Interactions	MALL-SCAL	

VPS4A	VPS4B	0.13337156	Physical Interactions	SMALL-SCALI	
VPS4A	VTA1	0.11032041	Physical Interactions	SMALL-SCALI	
LIN54	LIN9	0.076002054	Physical Interactions	ttlin-Harper-2	
LIN52	LIN9	0.14680636	Physical Interactions	ttlin-Harper-2	
LIN37	LIN9	0.073395744	Physical Interactions	ttlin-Harper-2	
LIN37	LIN54	0.13336256	Physical Interactions	ttlin-Harper-2	
LIN37	LIN52	0.2576045	Physical Interactions	ttlin-Harper-2	
MYBL1	LIN9	0.11752817	Physical Interactions	ttlin-Harper-2	
MYBL1	LIN54	0.21355268	Physical Interactions	ttlin-Harper-2	
MYBL1	LIN52	0.4125006	Physical Interactions	ttlin-Harper-2	
MYBL1	LIN37	0.2062294	Physical Interactions	ttlin-Harper-2	
L3MBTL2	LIN54	0.058416527	Physical Interactions	ttlin-Harper-2	
RBBP4	LIN9	0.058296047	Physical Interactions	ttlin-Harper-2	
RBBP4	LIN37	0.102293424	Physical Interactions	ttlin-Harper-2	
MYBL2	LIN9	0.21832462	Physical Interactions	ttlin-Harper-2	
MYBL2	LIN37	0.38309926	Physical Interactions	ttlin-Harper-2	
VTA1	VPS4B	0.17564446	Physical Interactions	ttlin-Harper-2	
VPS4A	VPS4B	0.24025449	Physical Interactions	ttlin-Harper-2	

VPS4A	VTA1	0.1835753	Physical Interactions	ttlin-Harper-2(	
CNOT4	CPEB3	0.0057032723	Shared protein domains	PFAM	
PLAGL2	OVOL1	0.004378151	Shared protein domains	PFAM	
PTBP3	CNOT4	0.005712123	Shared protein domains	PFAM	
MYBL2	MYBL1	0.09841619	Shared protein domains	PFAM	
VPS4A	VPS4B	0.04795295	Shared protein domains	PFAM	
